# Global Changes in Lipid Profiles of Mouse Cortex, Hippocampus, and Hypothalamus Upon p53 Knockout

**DOI:** 10.1038/srep36510

**Published:** 2016-11-07

**Authors:** Sang Tak Lee, Jong Cheol Lee, Jong Whi Kim, Soo Young Cho, Je Kyung Seong, Myeong Hee Moon

**Affiliations:** 1 Department of Chemistry, Yonsei University, Seoul 03722, Korea; 2 College of Veterinary Medicine, BK21 Program for Veterinary Science and Research, Institute of Veterinary Science, Seoul National University, Seoul 08826, Korea; 3 Korea Mouse Phenotyping Center (KMPC), Seoul 08826, Korea

## Abstract

Comprehensive lipidomic profiling in three different brain tissues (cortex, hippocampus, and hypothalamus) of mouse with p53 deficiency was performed by nanoflow liquid chromatography-tandem mass spectrometry (nLC-MS/MS) and the profile was compared with that of the wild type. p53 gene is a well-known tumour suppressor that prevents genome mutations that can cause cancers. More than 300 lipids (among 455 identified species), including phospholipids (PLs), sphingolipids, ceramides (Cers), and triacylglycerols (TAGs) were quantitatively analysed by selective reaction monitoring (SRM) of nanoflow ultrahigh performance liquid chromatography-electrospray ionization-tandem mass spectrometry (nUPLC-ESI-MS/MS). Among the three different neural tissues, hypothalamus demonstrated the most evident lipid profile changes upon p53 knockout. Alterations of PLs containing acyl chains of docosahexaenoic acid and arachidonic acid (highly enriched polyunsaturated fatty acids in the nervous system) were examined in relation to cell apoptosis upon p53 knockout. Comparison between sphingomyelins (SMs) and Cers showed that the conversion of SM to Cer did not effectively progress in the hypothalamus, resulting in the accumulation of SMs, possibly due to the inhibition of apoptosis caused by the lack of p53. Furthermore, TAGs were considerably decreased only in the hypothalamus, indicative of lipolysis that led to substantial weight loss of adipose tissue and muscles.

Lipidomics has recently gained considerable attention due to the various roles played by lipids, including as the structural components of cellular membranes, in energy storage, and as signal transduction messengers in the central and peripheral nervous systems^1^,^2^,^3^. Considerable alterations in lipid metabolism have been observed in various pathological events, such as coronary artery disease, cancer, Fabry disease, Gaucher disease, and diabetes, which suggests that lipids could be potential biomarkers of these diseases^4^,^5^,^6^,^7^,^8^.

Among diverse genetic mutations found in different types of cancers, the mutation in the p53 gene, also known as the “tumour suppressor gene”, is the most frequent^9^,^10^. Brain cancer or glioma is commonly classified into two types: astrocytoma, a benign tumour, and glioblastoma, the malignant tumour in adult patients^11^,^12^,^13^. p53 gene mutation has been reported in 70% of patients with astrocytoma, and in 11% and 67% of patients with primary and secondary glioblastomas, respectively, indicating p53 mutations are related to the development of brain tumour^14^,^15^. Besides tumour suppression, p53 is now known to play a more complicated role^16^,^17^. Especially, p53 is related to the metabolism involved in cancer development and prevention. In most cases of cancer, tumour development accompanies metabolic transformation, i.e., the change of the preferred energy production route from oxidative phosphorylation to glycolysis, resulting in limited oxygen supply followed by generation of considerable amount of reactive oxygen species. Thus, this metabolic transformation could activate the role of p53 through the activation of various p53-stimulating enzymes^18^,^19^. While studies on p53 have been conducted at the gene and protein levels, the effect of p53 gene mutation on the lipid profile has not been extensively investigated. Therefore, it is important to elucidate the functions of p53 in brain lipidomics with respect to the occurrence of glioma.

It is well-known that active cell-to-cell signalling takes place in the brain and that the brain controls the homeostasis of the whole biological system^20^,^21^. Neurodegenerative diseases, such as Alzheimer’s and Parkinson’s diseases, which threaten the quality of life of the elderly, affect normal brain functioning^22^,^23^. Docosahexaenoic acid (DHA) is a free fatty acid that is abundant in neural tissues and is critical in maintaining the normal functioning of the brain so that brain development during early childhood and mental well-being during senescence can be secured^24^. Decreased DHA level in the neural membrane is related to aging, and dietary supplementation of DHA helps restoring brain function^25^ and alleviating the impairment of cognitive functions, observed in patients with neurodegenerative diseases^26^. Among the lipids present in neural tissues, those with established roles in the brain are rare. Two classes of lipids, Cers and plasmalogens, are reported to be related to several phenomena in the brain. Cers that are produced by the cleavage of SM are important in cell signalling as a key mediator of apoptosis and are expected to be related to several age-related neurodegenerative diseases^27^. Plasmalogens are phospholipids (PLs) with a vinyl ether linkage in the sn-1 position of the glycerol backbone and are one of the most abundant types of lipids in the brain^28^. Although the functions of plasmalogens are relatively unknown compared to other lipids, a number of studies have demonstrated its relevance to several diseases, such as bronchopulmonary dysplasia (BPD), Neimann-Pick disease type C, Down syndrome, and Alzheimer’s disease^26^,^29^,^30^,^31^.

In this study, we performed comprehensive profiling of lipids in three different brain tissues (cortex, hippocampus, and hypothalamus) to investigate the distribution of lipids in these tissues and to evaluate their relevance to p53 deficiency. Non-targeted global search of lipids from different tissues was performed by determining the structure of individual lipids (up to 455 lipids, including PLs, SMs, Cers, and TAGs) from collision-induced dissociation (CID) experiments using nanoflow liquid chromatography-electrospray ionization-tandem mass spectrometry (nLC-ESI-MS/MS). More than half of the identified lipids from each tissue were selected for the high-throughput relative quantitation based on SRM, using nanoflow ultrahigh performance LC (nUPLC) with triple quadrupole mass spectrometry. Lipid distribution and relative amount of the lipids present in each type of brain tissue were evaluated and relative changes in individual lipids between wild type (WT) and p53 knockout (p53 KO) mice were statistically examined to elucidate the relationship between the functions of p53 and neural lipids.

## Results

### Lipid profiling in brain tissues

**Figure 1:**
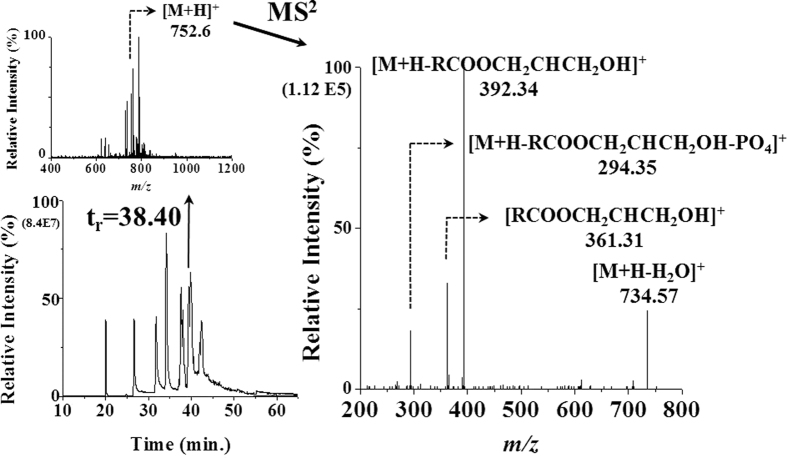
Base peak chromatogram (BPC) of the lipid extract of the wild type cortex tissue sample, the precursor MS spectra at t_r_ = 38.40 min, and CID spectra of m/z 752.6, [M+H]^+^, identified as 18:0p/20:4-PE plasmalogen by nLC-ESI-MS/MS analysis.

Identification of lipid molecular structures from brain tissues was carried out with each pooled tissue sample by nLC-ESI-MS/MS. Base peak chromatograms (BPCs) of cortex, hippocampus, and hypothalamus from WT and p53 KO mice obtained in negative ion mode are shown in Supplementary Fig. S1. In the same run condition, performance of nLC separation was demonstrated with lipid standards (see Supplementary Fig. S2) showing the separation of lipids with a broad hydrophobicity (from 18:1-lysophosphatidylcholine (LPC) to the most hydrophobic (54:1-TAG) as well as with a resolving capability of Phosphatidylethanolamine (PE)-plasmalogen (PEp; 18:0p/22:6-PE, peak 11) from 18:0/22:6-PE (peak 9) in the positive ion mode. While structural determinations of most lipid molecules were achieved with CID spectra in the same manner as reported in earlier studies^6^,^7^,^8^, determination of plasmalogen lipids was carried out by confirming the detection of unique product ions. Figure 1 shows BPC of the lipid extract of the wild type cortex tissue sample, the precursor scan MS spectra at t_r_ = 38.40 min of the BPC, and the CID spectra of an ion (m/z 752.6, [M+H]^+^), which was identified as 18:0p/20:4-PE from the detection of a characteristic product ion, [R_2_COOCH_2_CHCH_2_OH]^+^, at m/z 361.3 from the glycerol backbone with the acyl chain 20:4 at sn-2 position, a common fragment ion of PEp at m/z 392.3 representing the presence of 18:0p at sn-1, and an additional neutral loss of phosphoric acid (98 amu from m/z 392.3) at m/z 294.3.

From the non-targeted analysis, a total of 422, 425, and 455 lipids from cortex, hippocampus, and hypothalamus tissues, respectively, were identified, and the molecular structures are listed in Supplementary Table S1. While molecular structures of lipids were characterized with the location of acyl chains as well as structural isomers, including regioisomers, quantifications of PC, PE, and TAG species were made without differentiating these isomeric structures and therefore, chain structures of PC, PE, and TAG in Supplementary Table S1 are listed by the total numbers of carbons and double bonds, and retention times of every lipid classes, except TAG and PEp, are confirmed in negative ion mode. The detailed molecular structures of isomeric forms are listed in Supplementary Table S2. Since PC, PE, and TAG were quantified by the selective monitoring of the dominant product ions, [PCho+H]^+^ (m/z 183), [M+H-141]^+^, and [M+NH_4_-RCOONH_4_]^+^, respectively, differentiation of lipid isomers having different chain structures was not possible. The types of precursor ion and the corresponding product ion for SRM quantitation of each lipid class along with the total quantified number of lipids in each class are listed in Supplementary Table S3. Finally, 281 (cortex), 300 (hippocampus), and 312 (hypothalamus) lipids were quantified for each individual animal sample. Since quantification of lipids was carried out for a total 26 tissue samples in triplicate, an SRM time-table for a high-speed targeted analysis within 20 min per sample^32^ was utilized such that each individual lipid molecule was scanned during 2-min intervals (average bottom width of lipid peaks < 1 min). The quantitation results listed in Supplementary Table S1 represent the corrected peak area (relative to an internal standard (IS, 1 pmol) of each lipid class), the relative abundance (WT only), which was calculated by the peak area percentage (%) of each species within the corresponding lipid class, and the ratio (KO/WT) of corrected peak area of each lipid species. Lipid species that are underlined in Supplementary Table S1 represent the high-abundance species (defined as >100/number of lipids in each class). Supplementary Table S4 lists 16 IS lipids that have been selected with odd number of carbons in at least one acyl chain to compensate spectral fluctuations between runs.

### Quantitative insights in lipid changes upon p53 KO

**Figure 2:**
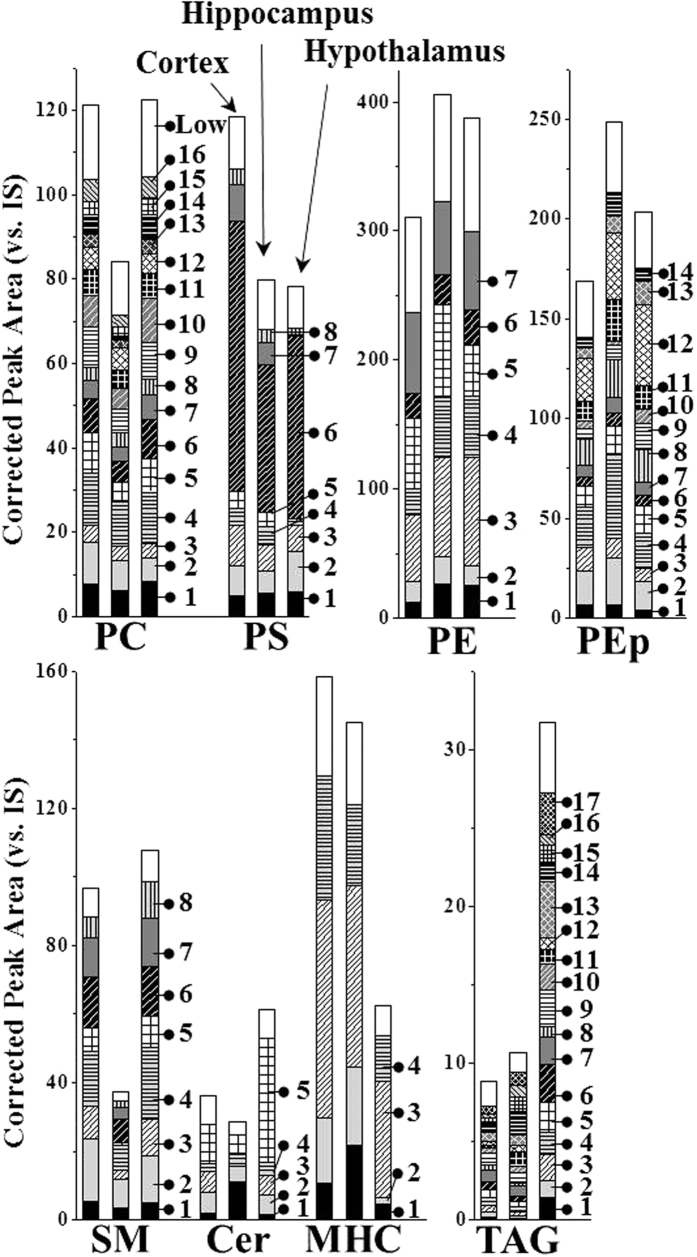
Comparison of the corrected peak area (vs. I.S.) of high-abundance lipid species in each class among different types of brain tissue. Molecular structures marked with individual numbers in each lipid class are listed in Supplementary Table S1. White bar marked with “low” represents the sum of the peaks area of remaining low-abundance lipid species in each class.

Distribution of each lipid among the different brain tissues was plotted by selecting 8 lipid classes showing remarkable differences among the tissues (Fig. 2). Lipid species represented by numbers were high-abundance species among each lipid class, and their molecular structures are listed in Supplementary Table S1. Bars marked with “low” in Fig. 2 represent the sum of peak areas of the remaining low-abundance species. Figure 2 shows the differences in the total lipid content of each class among three brain tissues as well as the distribution of individual species. The amounts of PC and SM were relatively lower in hippocampus than in the other two tissues, while PE and PEp were higher (the total amounts of each lipid class are listed in Supplementary Table S3). While the total level of monohexosylceramide (MHC) in hypothalamus was 2~3-fold lower than those in the other two tissues, the amount of TAG in hypothalamus was about 3-fold higher than that in the other two tissues. Variations in lipid composition were found among the different tissues; however, in this study the changes in the individual species due to the influence of p53 KO was examined at the molecular level.

**Figure 3:**
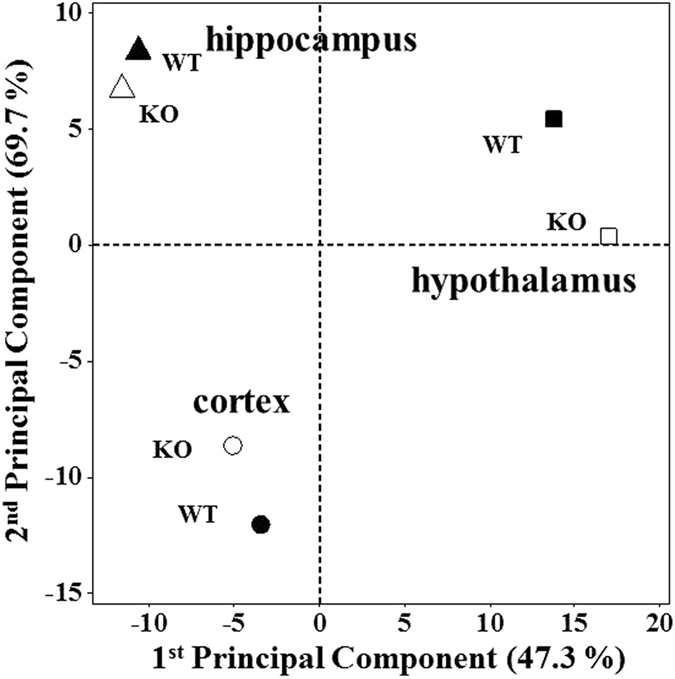
Score plot from the principal component analysis (PCA) of the entire lipid species in cortex, hippocampus, and hypothalamus demonstrates the statistical difference between wild type (WT, filled) and knockout (KO, empty).

The peak area ratios (KO/WT) in Supplementary Table S1 were expressed with both the data obtained from individual animal samples and the ratio obtained from pooled samples. By comparing the KO/WT ratio values between the individual and pooled measurements, their differences were <10%, indicating that they were not significantly different. Since amounts of hypothalamus tissue from each animal were very low in this study, data from pooled samples of hypothalamus were utilized to compare lipid changes with the individual results from the two other neural tissues. Comparison of the lipid profiles among the three tissues was performed with principal component analysis (PCA) for the quantified lipid species (Fig. 3), showing that lipids in hypothalamus exhibited considerable difference between KO and WT mice, while those in hippocampus showed the least changes. In Supplementary Fig. S3, heat maps representing the changes in the relative amounts of 324 lipid species are plotted for cortex and hippocampus only (a pooled sample was analysed for hypothalamus). Among the quantified lipids in Supplementary Table S1, lipids showing a significant change (>2-fold changes between KO and WT, P < 0.01) are listed in Table 1 and depicted in bold. The numbers of species showing significant changes were 8 for hippocampus, 24 for cortex, and 37 for hypothalamus, supporting the PCA results. It showed that significant decreases in PEp and TAG species were found in the hypothalamus, while in cortex, PC and sphingolipid species were lowered, but PIs were increased. Among these species, the KO/WT ratio of highly abundant species showing >2-fold change (underlined in Table 1) has been plotted in Supplementary Fig. S4, indicating that most of the high abundance species were down-regulated in the three neural tissues with the p53 KO. Abundant PEp species in cortex were increased by about 2-fold, but all PEp species in hypothalamus were decreased. However, d18:1/24:1-SM showed the opposite trend: a decrease in cortex, but an increase in hypothalamus. Most TAG species exhibited decreases in hypothalamus and a few in hippocampus, but they were not significantly changed in the cortex.

The role of p53 gene has been known to prevent the development of tumours through cell apoptosis^33^. Several studies showed that apoptosis can be altered in several chronic diseases (cardiovascular, neurodegenerative, immune, and inflammatory diseases, including cancer), which can be inhibited by the beneficial effect of polyunsaturated fatty acids (PUFAs) that were highly enriched in nervous system^34^,^35^. Among PUFAs, free unesterified arachidonic acid (AA, 20:4) in cells was reported to inhibit cell growth and induce apoptosis, and DHA (22:6) was reported to exert effects in inhibiting the development of the above stated diseases by inducing apoptotic process^7^,^8^,^36^,^37^. Lipids containing acyl chains of 22:6 or 20:4 in PC, PI, PE, and PEp classes were plotted (Supplementary Fig. S5) to compare their relative changes upon p53 KO. PC species containing these PUFAs were found to increase by about 50% in hippocampus, while they were not changed in the cortex and hypothalamus. PIs exhibited an opposite trend to PCs. PE and PEp containing 22:6 or 20:4 acyl chains showed increases in the cortex, but decreased in hypothalamus, while these were not changed in hippocampus. Moreover, PEp containing 22:6 (16:1p, 18:0p, and 18:1p in sn-1) were significantly lowered in hypothalamus.

**Figure 4:**
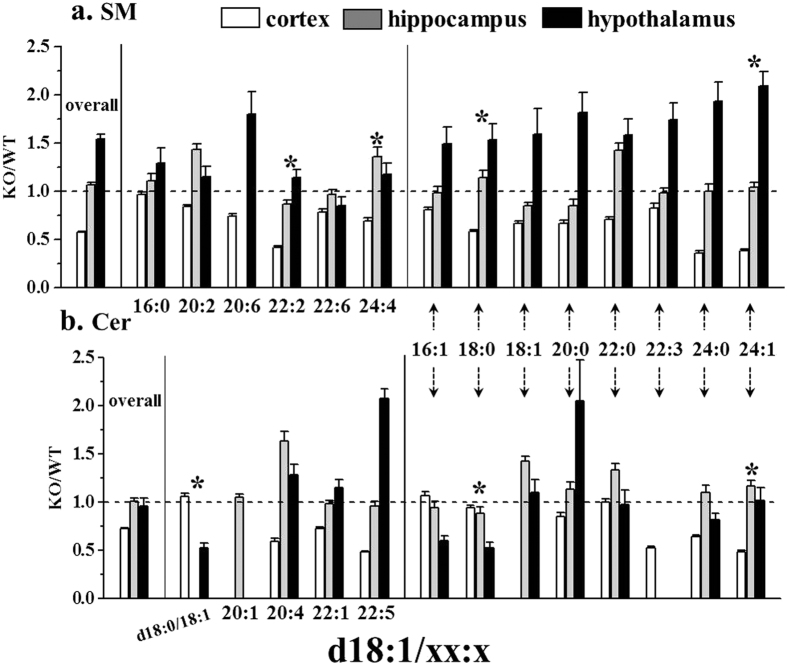
Changes in the amount of total and individual lipid species of SM and Cer groups indicating the dysfunctional SM metabolism in cortex and hypothalamus.

Sphingolipids, including SM and Cer, have been known to play messenger roles in regulating cell proliferation, survival, and apoptosis^38^,^39^. Since Cer can be produced from the degradation of SM by sphingomyelinases as one of the several metabolic pathways to generate Cer and Cer mediates inhibition of cell growth and induction of apoptosis^40^, the up- and down-regulations of Cer and SM may provide an important insight in understanding the mechanism of cell apoptosis. Figure 4 shows the relative changes between the overall levels of SM and Cer upon p53 KO, and the changes in individual molecules. While the overall amounts of SM and Cer did not change in the hippocampus upon p53 KO, they were reduced in cortex. However, in hypothalamus, the overall SM level increased (most SM species in Fig. 4a) about 50%, but the overall Cer level was not altered. Among the 14 SMs and 13 Cers in Fig. 4, 8 chain structures (16:1, 18:0, 18:1, 20:0, 22:0, 22:3, 24:0, and 24:1 for the sn-2 chain type of d18:1/xx:x) were found in both, SM and Cer, on the right half of the plots and most of these eight SMs exhibited decreases in the cortex, but Cer did not increase. However, levels of the 8 SMs increased in hypothalamus, but the corresponding Cers did not decrease as much as the change in SMs, except d18:1/16:1 and d18:1/18:0, of which the latter was the highest abundance species among Cers.

**Figure 5:**
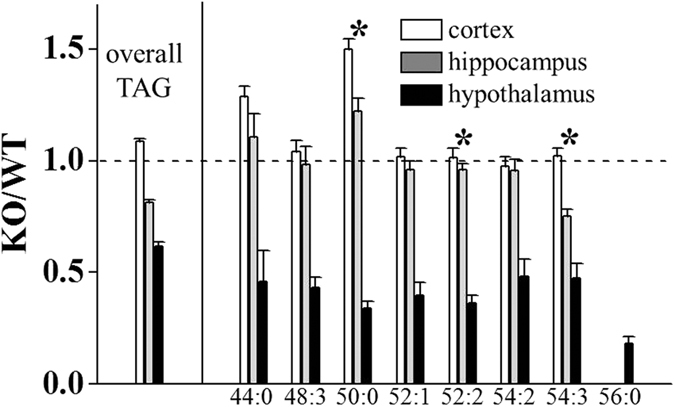
Changes in the TAG species with >2-fold change in hypothalamus are depicted with their relative changes in cortex and hippocampus.

One of the remarkable changes observed among lipid classes on p53 KO was the significant decreases (>2-fold) of most TAG levels in hypothalamus, whereas those in cortex and hippocampus were not altered, except 50:0-TAG (which exhibited an ~50% increase in cortex), as shown in Fig. 5. These results support that degradation of TAG can be enhanced in the hypothalamus of brain tissue by the p53 KO, while cortex and hippocampus were not affected.

**Figure 6:**
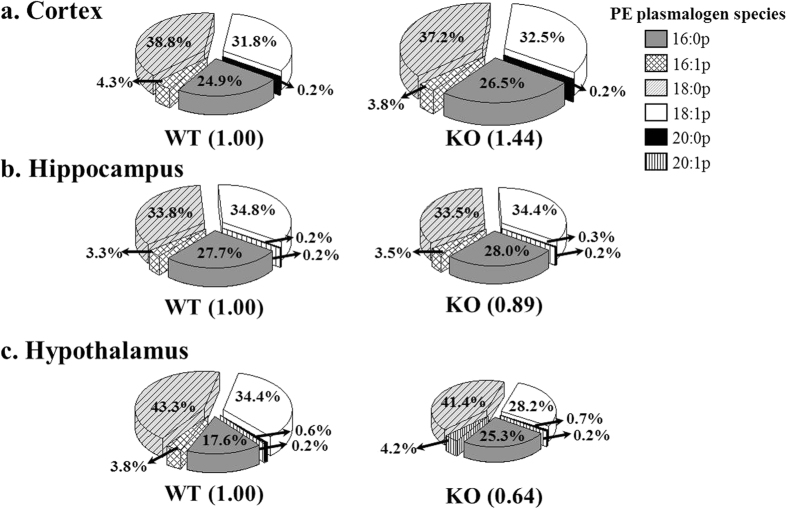
Pie chart representing the relative change in PEp and their chain composition in WT and KO. The numbers in the parentheses below each pie plot denote the total level of PEp species relative to that in WT, which was set to 1.

Plasmalogens, enriched in the nervous system, showed clear differences in the hypothalamus of p53 KO mice in this study. Figure 6 shows the comparison of the relative amounts of PEp species plotted against the 6 common sn-1 acyl chain structures (16:0p, 16:1p, 18:0p, 18:1p, 20:0p, and 20:1p in Supplementary Table S1) between WT and KO. The number in the parentheses for WT was set to 1.00 to compare the relative change in the entire amount of PEp in KO, and the percentage values in the pie chart represent the occupancy of each chain type based on the relative peak areas (vs. 17:0/17:0-PE as IS) of individual species. While the total amount of PEp was increased by 44% in cortex or decreased by 11% in hippocampus with KO, compositional abundances based on each chain type were not much altered as shown in Fig. 6a,b. However, the total PEp amount was decreased by 36% in hypothalamus in Fig. 6c and showed the variation in the amounts of individual chain types: noticeable decrease in PEp with 16:0p and 18:1p.

## Discussion

Comprehensive lipid profiles of cortex, hippocampus, and hypothalamus of mouse were generated and the influence of the p53 KO on brain lipids (312 quantified from 455 identified lipids) was analysed by nUPLC-ESI-MS/MS. The lipid profiles of the three neighbouring brain tissues with different neuronal functions in brain differed substantially in terms of relative abundance of the lipids, and the influence of p53 deficiency on the lipid profiles were independent of tissue type. Recent studies indicated that p53 regulated metabolic pathways, such as glucose metabolism and oxidative phosphorylation, leading to tumour related metabolic changes in lipids^41^,^42^. In the present study, most lipids identified in hippocampus were not noticeably influenced by p53 KO, while various types of lipids from the cortex and hypothalamus exhibited substantial changes, suggesting that the effect of p53 KO on different brain sub-regions was independent of tissue type.

Significant decreases in TAG levels were observed only in the hypothalamus, but not in the cortex and hippocampus. Decrease in TAG levels may be associated with lipolysis, the increased degradation of TAGs by triglyceride lipase^43^,^44^. Lipolysis is known to initiate and/or progress cancer-associated cachexia, which is a multifactorial devastating syndrome common in most cancer patients, leading to substantial weight loss, mainly by the loss of adipose and muscle masses^37^,^45^,^46^,^47^. In addition, it has been reported that p53 inhibits lipid accumulation by regulating the pentose phosphate pathway (PPP)^48^.

In the case of the 6 PEp species, 44% increase in the total amounts was found in cortex without a change in compositional abundances of 6 sn-1 chain types, and 36% decrease was observed along with a compositional change in the hypothalamus. Although the roles of plasmalogens have not been clearly understood, it was reported that plasmalogens protected cells against damages by reactive oxygen species^49^ and the reduced levels of plasmalogens were associated with Alzheimer’s disease and adrenoleukodystrophy^50^,^51^.

For SM and Cer species sharing 8 common chain structures, SMs were remarkably decreased in cortex and accumulated in hypothalamus, whereas Cers matching the same chains with SMs remained unchanged in both tissues. According to a study on the roles of p53 to induce Cer accumulation by the activation of sphingomyelinases^52^, it may be explained that the conversion of SM to Cer did not effectively progress in hypothalamus, resulting in the accumulation of SMs due to the inhibition of apoptosis caused by p53 deficiency. However, it was determined that the formation of Cer from SM may not be the only pathway in cortex. A report on MCF7 breast carcinoma cells showed that p53 induced up-regulation of neural sphingomyelinase-2, resulting in an increase in Cers.

In the case of PLs containing acyl chains of 22:6 or 20:4, PIs were increased in both, cortex and hypothalamus, but PEs (including PEp) exhibited increases in cortex but decreases in hypothalamus. Decrease in DHA level in the adult brain was reported in pathophysiological conditions caused by aging, Alzheimer’s disease, and alcohol exposure^53^,^54^,^55^. It may be explained that in p53 KO mice, the release of AA and DHA from phospholipids was tissue-dependent: abundant PCs containing these AA or DHA were accumulated in hippocampus, PIs, PEs, and PEp were accumulated in cortex, but most PEs and PEps with either of the two PUFAs were destroyed in hypothalamus.

## Methods

### Materials and Reagents

For the selection of an optimized run condition for tissue lipid analysis, the following 40 standard lipids from Avanti Polar Lipids, Inc. (Alabaster, AL, USA) and Matreya, LLC. (Pleasant Gap, PA, USA) were utilized: 17:0-lysophosphatidylcholine (LPC), 18:1-LPC, 13:0/13:0-phosphatidylcholine (PC), 18:1/18:0-PC, 20:0/20:0-PC, 14:0-lysophosphatidylethanolamine (LPE), 17:1-LPE, 18:0-LPE, 14:0/14:0-phosphatidylethanolamine (PE), 17:0/17:0-PE, 18:0/22:6-PE, 14:0-lysophosphatidylglycerol (LPG), 17:1-LPG, 12:0/12:0-phosphatidylglycerol (PG), 15:0/15:0-PG, 17:1-lysophosphatidylserine (LPS), 12:0/12:0-phosphatidylserine (PS), 17:0/20:4-PS, 16:0/18:2-phosphatidylinositol (PI), 17:0/20:4-PI, 12:0-lysophosphatidic acid (LPA), 17:0-LPA, 14:0/14:0-phosphatidic acid (PA), 17:0/17:0-PA, d18:1/12:0-SM, d18:1/17:0-SM, d18:1/18:0-SM, d18:1/17:0-MHC, d18:1/18:0-MHC, d18:1/16:0-dihexosylceramide (DHC), d18:1/17:0-DHC, d18:1/12:0-Cer, d18:1/14:0-Cer, d18:1/17:0-Cer, d18:1/22:0-Cer, d18:1/17:0-sulfatide (ST), d18:1/24:0-ST, 18:0/18:1-diacylglycerol (DAG), 17:0/17:1/17:0 D_5_-TAG, 54:1 (18:0/18:1/18:0)-TAG. Lipids with odd numbered fatty acyl chains were used as internal standards for the quantitation of each targeted class of lipids. Solvents (water, acetonitrile, methanol, and isopropanol) used for the mobile phases were HPLC-grade, purchased from Avantor Performance Materials (Center Valley, PA, USA). NH_4_HCO_3_, NH_4_OH, and methyl *tert*-butyl ether (MTBE) were purchased from Sigma-Aldrich (St. Louis, MO, USA). Fused silica capillary columns with inner diameters of 20, 50, 75, or 100 μm (outer diameters are the same for all, 360 μm) were purchased from Polymicro Technology, LLC (Phoenix, AZ, USA). The packing materials used for LC columns were Watchers^®^ ODS-P C-18 particles (3 μm and 100 Å) purchased from Isu Industry Corp. (Seoul, Korea) and 1.7 μm ethylene bridged hybrid (BEH) particles unpacked from XBridge^®^ BEH C18 column (1.7 μm, 2.1 mm × 100 mm) from Waters (Milford, MA, USA).

### Animals

Six male p53 KO mice purchased from the Jackson Laboratory (Bar Harbor, ME) and six C57BL/6N mice (as WT) obtained from Korea Research Institute of Bioscience and Biotechnology (Daejeon, Korea) were maintained in the animal facility at Seoul National University. Brain samples were paired from WT and p53 KO mouse. In order to reduce the difference among samples when using C57 BL/6N genetic background mice, litter mates from same parents (p53−/+ intercross breeding) were used as wild type controls compared with p53 KO mouse. Animals were housed at 24 ± 2 °C with a 12-h light/dark cycle and fed with a normal diet, NIH-31 from Zeigler Bros, Inc. (Gardners, PA, USA) *ad libitum* with tap water. Animals were humanely sacrificed by CO_2_ exposure. Animal experiments followed the “Guide for Animal Experiments” edited by Korean Academy of Medical Sciences under the approval by the Institutional Animal Care and Use Committee (IACUC) of Seoul National University.

### Lipid Extraction from Brain Tissues

A total of 36 tissue samples (from cortex, hippocampus, and hypothalamus from six WT and six p53 KO mice) were provided in a dried condition from the Korea Mouse Phenotyping Center. Each brain tissue sample was lyophilized and crushed into powder. For the global identification of lipids from each tissue type, 333–335 μg of individual powdered samples was mixed together to prepare a 2 mg pooled WT and p53 KO samples for cortex, hippocampus, and hypothalamus. For targeted quantitative analysis, 2 mg of tissue from each individual sample was taken, except for hypothalamus (where the weight of individual tissue sample was less than 2 mg and pooled sample of 2 mg was prepared by adding 335–335 μg of six individual samples) to get 6 cortex, 6 hippocampus, and 1 pooled hypothalamus samples from WT and KO. Lipid extraction from tissue sample was done by the modified Folch method with MTBE/CH_3_OH^56^. Powdered tissue sample was dissolved in 300 μL of CH_3_OH and sonicated in bath sonicator for 10 min at 48 °C. After adding 1,000 μL of MTBE, sample mixture was incubated overnight at 48 °C with constant stirring at 750 rpm. The latter incubation procedure was added in this study to assure the extraction of SM and Cers, which tend to have a high transition temperature and are key players of cell signalling related to cell apoptosis^57^,^58^,^59^. Each incubated sample mixture was cooled down and 250 μL of H_2_O was added, followed by centrifugation at 1000 × *g* for 10 min. After the upper organic layer was transferred to a separate test tube, 300 μL of CH_3_OH was added to the aquatic layer to ensure more thorough extraction of lipids, followed by sonication for 2 min and centrifugation at 1000 × *g* for 10 min. The upper organic layer was combined with the previous organic extract. Lipid extract was then lyophilized overnight, weighed and re-dissolved in CHCl_3_:CH_3_OH (3:7, v/v) to make the concentration to 20 μg/μL. Dissolved lipid extract was transferred to a glass container for storage at −87 °C. For nLC–ESI–MS/MS analysis, each sample was diluted to a concentration of 2.5 μg/μL with CH_3_OH:H_2_O (9:1, v/v).

### nLC–ESI–MS/MS

Two nLC-ESI-MS/MS systems were employed in this study: a model 1200 capillary HPLC pump with autosampler from Agilent Technologies (Palo Alto, CA, USA) hyphenated with LTQ Velos ion trap mass spectrometer from Thermo Scientific (San Jose, CA, USA) for the non-targeted lipid identification and a nanoACQUITY UPLC system with an autosampler from Waters (Milford, MA, USA) coupled to a TSQ Vantage triple-stage quadrupole MS system from Thermo Scientific for high speed lipid quantitation using selected reaction monitoring (SRM). Analytical columns were prepared with fused silica capillary (360 μm O.D.) by pulling the one end with flame to make a sharp needle which acts as a direct emitter for ESI. Mobile phases for binary gradient elution were the same for both systems: A with H_2_O:CH_3_CN (9:1, v/v) and B with CH_3_OH:CH_3_CN:isopropanol (2:2:6, v/v). Both were added with a mixed modifier (5 mM NH_4_HCO_3_ and 0.05% NH_4_OH). Column preparation procedure and gradient elution method are explained in Supplementary Information.

For non-targeted lipid identification, each lipid extract was loaded to the column with mobile phase A at 800 nL/min for 10 min with the switching valve off. Then the pump flow rate was raised to 10 μL/min with the switching valve on (flow split to vent capillary at the microcross), resulting in 300 nL/min entering the column. This was to reduce dwell time. Injection amount of the lipid extract was 4 μL (equivalent to 10 μg) including 1 pmol of two internal standards (IS, 13:0/13:0-PC for positive ion mode and 15:0/15:0-PG for negative mode). Three repeated runs were made for each sample in both positive and negative ion modes with the MS detection range of precursor ion at m/z 400~1200 at 3.0 kV of ESI. Data-dependent CID experiment of precursor ion was accomplished by applying 40% of normalized collision energy. Structures of lipid species in each brain tissue were elucidated by matching CID spectrum with theoretical spectrum, which was done by algorithm-based search software called LiPilot developed previously^60^.

For targeted quantitation, each lipid extract sample was spiked with an IS mixture containing 16 lipid standards of different classes with odd numbered acyl chain (listed in Supplementary Table S1) at a concentration of 250 fmol/μL for each IS. Injection amount for the 26 lipid extract samples was 4 μL which contained 10 μg of tissue sample together with 1 pmol of each IS. Each tissue sample was analyzed in triplicate. PCs, PEs, PE plasmalogens (pPEs), SMs, Cers, MHCs, STs and TAGs were detected in positive ion mode and PGs, PIs, PAs and PSs in negative ion mode. Detection of ions in both positive and negative ion modes was automatically switched by MS at 3.0 kV of ESI, with scan width at m/z 1.50, and scan time at 0.001 s. SRM quantitation of nearly 300 lipids was achieved by applying different collision energy to each lipid class in Supplementary Table S3 along with the type of precursor ion and each corresponding product ion for 18 lipid classes. Two statistical analyses of data, principal component analysis by Minitab 17 software (http://www.minitab.com), and Mann-Whitney U test by IBM SPSS software from IBM Corp (Armonk, Ny, USA), was accomplished.

## Additional Information

**How to cite this article**: Lee, S. T. *et al*. Global Changes in Lipid Profiles of Mouse Cortex, Hippocampus, and Hypothalamus Upon p53 Knockout. *Sci. Rep.* **6**, 36510; doi: 10.1038/srep36510 (2016).

**Publisher’s note:** Springer Nature remains neutral with regard to jurisdictional claims in published maps and institutional affiliations.

## Supplementary Material

Supplementary Information

## Figures and Tables

**Table 1:** Lipid species with >2-fold change on p53 knockout (P < 0.01 marked in bold) are expressed with the relative peak areas and standard deviation (triplicate measurements) of lipid species from cortex (n = 6), hippocampus (n = 6), and hypothalamus (pooled).

Class	Species	m/z	Cortex (n = 6)	Hippocampus (n = 6)	Hypothalamus (pooled)	Class	Species	m/z	Cortex (n = 6)	Hippocampus (n = 6)	Hypothalamus (pooled)
**PC**	22:4	572.3	1.07 ± 0.04	0.88 ± 0.04	2.02 ± 0.13	**PI**	18:0/20:3	887.7	**2.08 ± 0.09**	0.61 ± 0.03	1.40 ± 0.04
	38:1	816.5	**0.33 ± 0.02**	1.25 ± 0.07	0.71 ± 0.07		20:4/20:4	905.7	1.70 ± 0.05	**0.50 ± 0.01**	**2.00 ± 0.13**
	38:0	818.5	**0.36 ± 0.02**	1.42 ± 0.07	0.98 ± 0.09		20:4/20:3	907.7	**3.13 ± 0.26**	0.91 ± 0.06	1.90 ± 0.13
	40:2	842.5	**0.42 ± 0.03**	1.12 ± 0.05	0.94 ± 0.07		18:0/22:6	909.7	**2.02 ± 0.06**	0.95 ± 0.06	1.34 ± 0.02
	40:1	844.5	**0.21 ± 0.01**	1.65 ± 0.07	0.60 ± 0.09		20:1/20:4	911.7	**2.67 ± 0.11**	0.77 ± 0.03	1.08 ± 0.08
	40:0	846.5	**0.31 ± 0.01**	**2.00 ± 0.21**	0.61 ± 0.04	**LPA**	18:0	437.5	**0.48 ± 0.05**	1.10 ± 0.05	0.98 ± 0.08
	42:2	870.5	**0.31 ± 0.01**	**2.07 ± 0.16**	0.86 ± 0.09		22:6	481.5	1.05 ± 0.05	1.20 ± 0.04	**3.98 ± 0.31**
	42:1	872.5	**0.42 ± 0.03**	1.73 ± 0.17	0.67 ± 0.09	**PA**	16:0/22:4	723.5	**0.47 ± 0.03**	N.Q.	1.22 ± 0.12
**PE**	16:1	452.3	N.D.	0.83 ± 0.03	**2.48 ± 0.27**	**LPG**	14:0	455.5	1.73 ± 0.31	**0.06 ± 0.01**	0.89 ± 0.10
	22:6	526.3	1.05 ± 0.04	1.04 ± 0.04	**3.10 ± 0.36**	**PG**	18:0/16:0	749.6	**2.33 ± 0.18**	0.62 ± 0.03	0.95 ± 0.03
	36:5	738.5	1.53 ± 0.05	1.05 ± 0.07	**0.34 ± 0.03**		22:6/22:5	867.6	**0.47 ± 0.03**	1.31 ± 0.14	1.03 ± 0.08
	38:6	764.5	1.64 ± 0.08	0.86 ± 0.05	**0.34 ± 0.03**	**TAG**	44:0	768.7	1.29 ± 0.04	1.11 ± 0.10	**0.46 ± 0.14**
	38:1	774.5	1.08 ± 0.05	1.03 ± 0.11	**0.37 ± 0.04**		48:3	818.7	1.04 ± 0.05	0.98 ± 0.08	**0.43 ± 0.05**
	40:6	792.5	1.04 ± 0.05	1.02 ± 0.03	**0.50 ± 0.02**		50:0	852.7	1.50 ± 0.05	1.22 ± 0.06	**0.34 ± 0.03**
**PEp**	16:0p/20:4	724.6	0.73 ± 0.04	1.00 ± 0.06	**0.39 ± 0.04**		52:4	872.7	1.56 ± 0.13	**0.50 ± 0.03**	0.58 ± 0.05
	16:0p/22:6	748.6	**2.02 ± 0.08**	0.83 ± 0.04	1.02 ± 0.10		52:2	876.7	1.02 ± 0.04	0.96 ± 0.03	**0.36 ± 0.03**
	18:1p/20:4	750.6	0.99 ± 0.02	0.67 ± 0.05	**0.47 ± 0.03**		52:1	878.7	1.02 ± 0.04	0.96 ± 0.04	**0.40 ± 0.06**
	18:1p/20:1	756.6	1.96 ± 0.05	0.89 ± 0.07	**0.36 ± 0.03**		54:6	896.7	1.19 ± 0.06	**0.39 ± 0.03**	0.81 ± 0.05
	18:0p/20:1	758.6	0.73 ± 0.02	0.99 ± 0.04	**0.45 ± 0.05**		54:3	902.7	1.02 ± 0.03	0.75 ± 0.03	**0.47 ± 0.06**
	18:0p/20:0	760.6	N.Q.	0.92 ± 0.04	**0.33 ± 0.07**		54:2	904.7	0.98 ± 0.04	0.96 ± 0.05	**0.48 ± 0.08**
	18:1p/22:6	774.6	**2.05 ± 0.07**	1.00 ± 0.07	**0.49 ± 0.02**		56:0	936.7	N.D.	N.Q.	**0.18 ± 0.03**
	16:0p/24:4	780.6	N.D.	0.98 ± 0.03	**0.37 ± 0.04**	**SM**	d18:1/22:2	783.5	**0.42 ± 0.01**	0.87 ± 0.03	1.14 ± 0.08
	18:1p/22:1	784.6	**0.45 ± 0.02**	1.01 ± 0.06	**0.46 ± 0.05**		d18:1/24:1	813.5	**0.38 ± 0.01**	1.04 ± 0.05	**2.09 ± 0.15**
	18:0p/22:1	786.6	0.51 ± 0.03	N.D.	**0.49 ± 0.06**		d18:1/24:0	815.5	0.36 ± 0.02	1.00 ± 0.07	1.94 ± 0.20
	20:0p/20:1	786.6	N.Q.	1.14 ± 0.08	**0.26 ± 0.02**	**Cer**	d18:1/20:0	594.5	0.85 ± 0.04	1.14 ± 0.07	**2.05 ± 0.42**
	18:0p/24:5	806.6	N.Q.	0.93 ± 0.04	**0.27 ± 0.03**		d18:1/22:5	612.5	**0.49 ± 0.01**	0.96 ± 0.05	**2.08 ± 0.10**
	18:1p/24:4	806.6	N.Q.	0.90 ± 0.04	**0.27 ± 0.02**		d18:1/24:1	648.5	**0.48 ± 0.01**	1.17 ± 0.05	1.01 ± 0.14
	20:1p/22:4	806.6	N.Q.	1.11 ± 0.10	**0.42 ± 0.06**	**MHC**	d18:1/18:0	728.5	0.91 ± 0.02	1.38 ± 0.06	**2.08 ± 0.38**
**LPS**	22:4	572.5	1.29 ± 0.07	1.18 ± 0.07	**2.12 ± 0.30**		d18:1/20:1	754.5	0.83 ± 0.02	**2.06 ± 0.13**	1.31 ± 0.14
	24:0	608.5	0.87 ± 0.04	0.98 ± 0.05	**2.12 ± 0.22**		d18:1/22:0	784.5	1.69 ± 0.05	**2.09 ± 0.14**	1.97 ± 0.16
**PS**	18:0/20:2	814.5	0.99 ± 0.06	0.96 ± 0.08	**2.05 ± 0.21**	Total	>2-fold, P < 0.01		24	8	37
	20:4/22:6	854.5	**0.50** ± **0.04**	1.37 ± 0.07	1.69 ± 0.08		#of quantified		281	300	312
	22:6/20:0	862.5	N.D.	N.D.	**2.53 ± 0.45**		#of identified		422	425	455

Underlined species are abundant in each class of lipids.
