# Supplementary material for: Global Changes in Lipid Profiles of Mouse Cortex, Hippocampus, and Hypothalamus Upon p53 Knockout
Source: Sci Rep. 2016 Nov 7;6:36510. doi: 10.1038/srep36510 (PMC5098149; doi:10.1038/srep36510)
Supplement: Supplementary Information [file srep36510-s1.docx]

Supplementary Information

Global Changes in Lipid Profiles of Mouse Cortex, Hippocampus, and Hypothalamus Upon p53 Knockout

Sang Tak Lee, ^1^ Jong Cheol Lee,^1^ Jong Whi Kim,^2,3^ Soo Young Cho, ^2,3^ Je Kyung Seong, ^2,3*^ and Myeong Hee Moon^1,*^

^1^Department of Chemistry, Yonsei University, Seoul 03722, Korea

^2^College of Veterinary Medicine, BK21 Program for Veterinary Science and Research Institute of Veterinary Science, Seoul National University, Seoul 08826, Korea

^3^Korea Mouse Phenotyping Center (KMPC), Seoul 08826, Korea.

Table of Contents

Column preparation and gradient elution 2

Figure S1 3

Figure S2 4

Figure S3 5

Figure S4 6

Figure S5 7

Table S1 8

Table S2 23

Table S3 26

Table S4 28

***Column preparation and gradient elution methods***

Columns were packed with 3 μm-100 Å Watchers® ODS-P C-18 particles for 7 cm in 75 µm I.D. capillary for the use of non-targeted analysis under nitrogen gas at 1000 psi. For targeted quantitation, the capillary tip was packed for with 3-μm C-18 Watchers particles for the first 0.5 cm from the end of pulled needle tip to make self-assembled frit, followed by packing 1.7 μm BEH particles for 6.5 cm in a 100 µm I.D. capillary. Each capillary column was connected with the capillary tubing from pump by PEEK microcross from IDEX (Oak Harbor, WA, USA). The other two ports on the side of microcross were connected to Pt wire for applying ESI voltage and to a vent capillary (20 µm I.D. and 360 µm O.D.) for splitting the pump flow.

Gradient method for non-targeted qualitative analysis began by increasing the mobile phase B from 0 to 80% within 10 min and 100% over next 15 min., and it was maintained for 45 min to wash off the remaining lipids in the column. Then the column was re-equilibrated by increasing the mobile phase A to 100% and maintained for 20 min before the next run. Gradient for targeted analysis was made as follows: sample loading was made at 800 nL/min with mobile phase A for 10 min followed by ramping up the follow rate to 12 μL/min with the split valve open to make a final flow rate to 300 nL/min. Gradient began by increasing the mobile phase B from 0 to 50% over the 1.5 min., to 80% for the next 2.5 min, further to 100% for 5 min, and then maintaining at 100% for 11 min. After completion of the elution, 5 min of re-equilibration was achieved with the mobile phase A


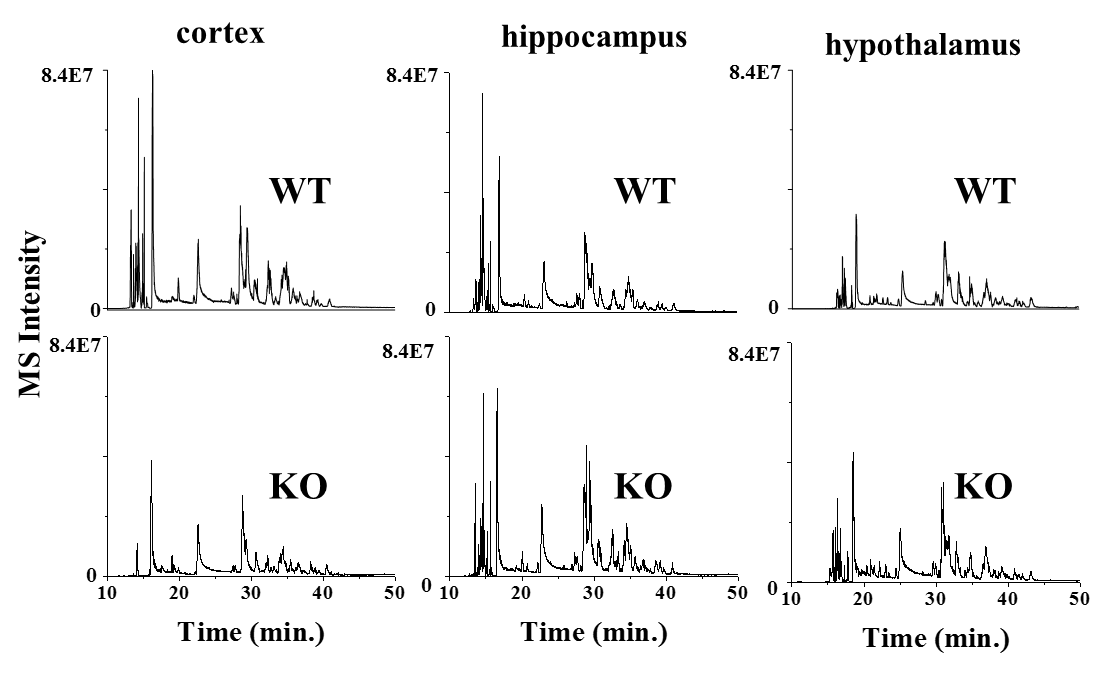


Figure S1. Base peak chromatograms (BPC) of the three different neural tissues from WT andp53 KO mice obtained in negative ion mode of nLC-ESI-MS/MS.


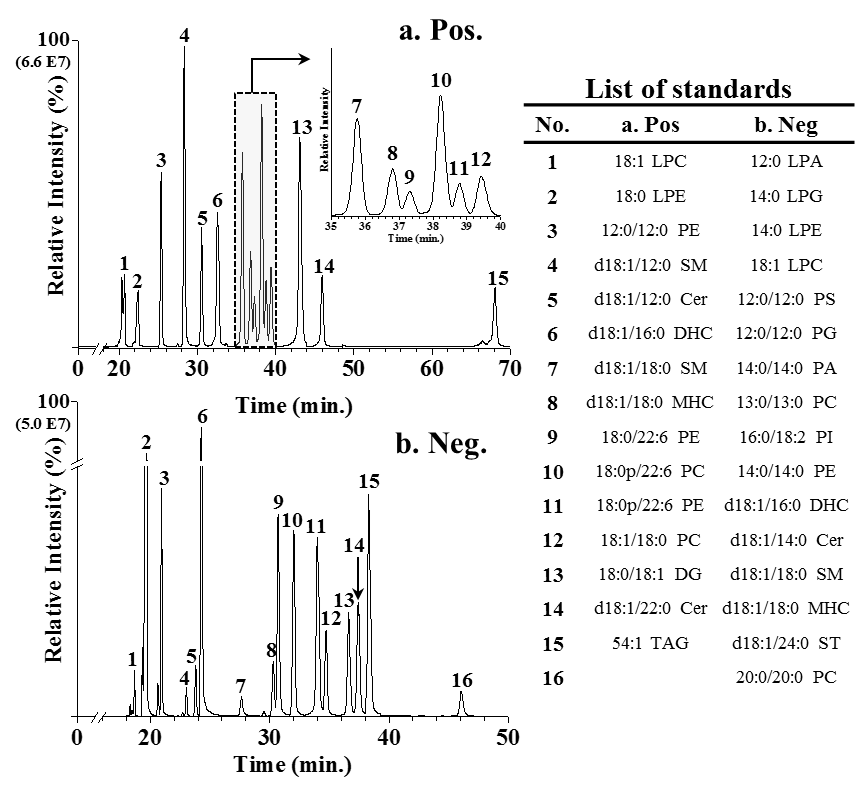


**Figure S2.** Base peak chromatogram (BPC) of 27 lipid standards in a) positive and b) negative ion modes


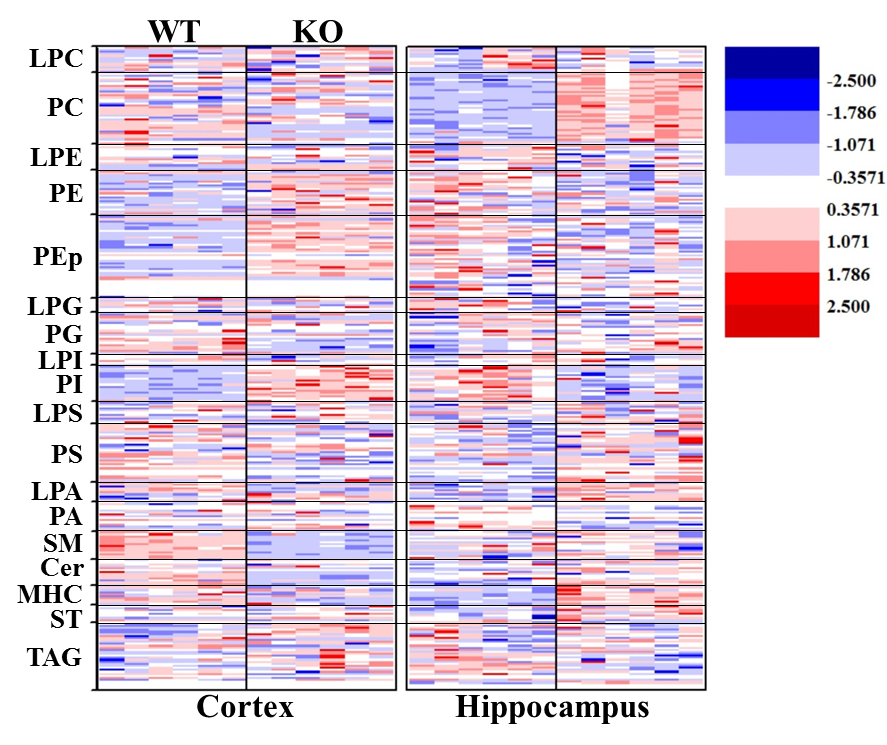


**Figure S3.** Heat map representing 324 lipid species in cortex and hippocampus of 6 individual wild type and knockout mice


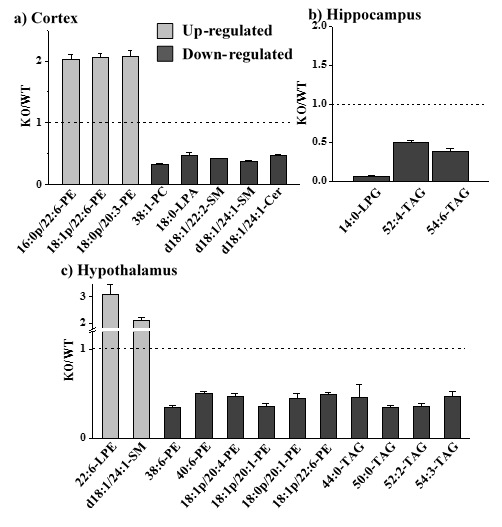


**Figure S4.** Ratio (KO/WT) of high-abundance lipid species in each class with >2-fold change (P < 0.01). Up-regulated species are depicted in light grey and down-regulated species in dark grey.


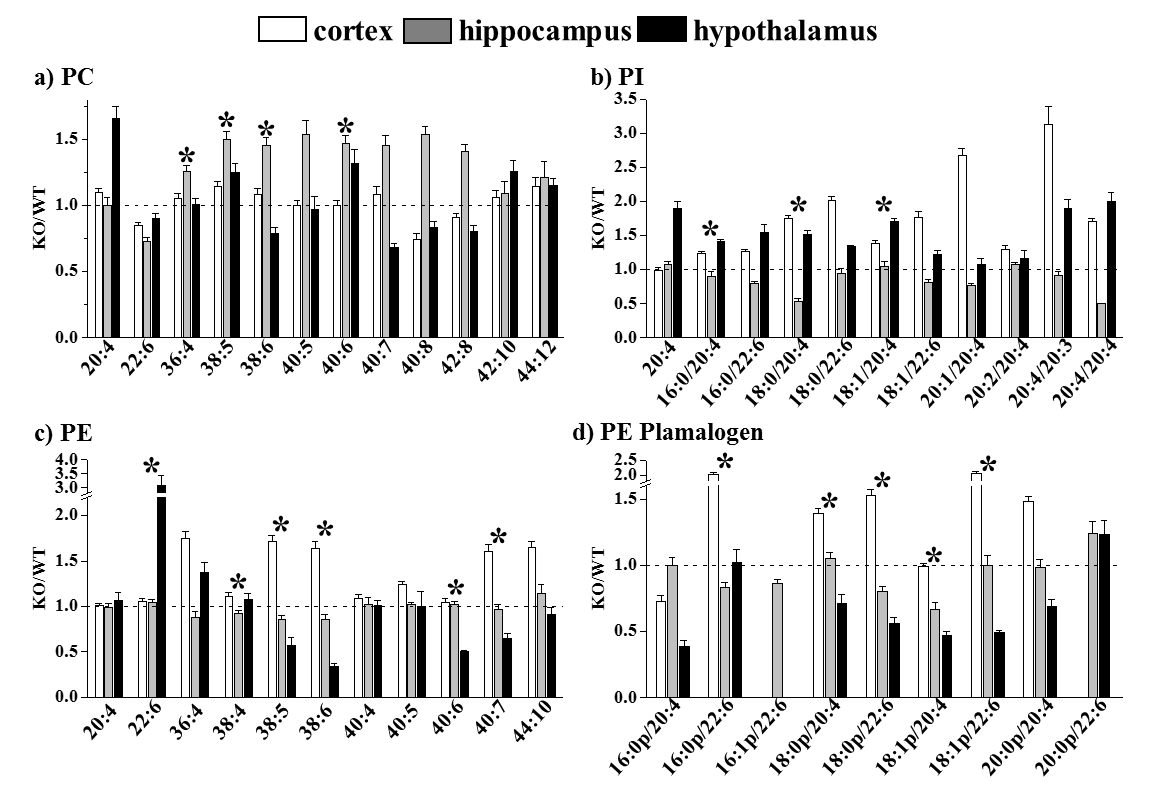


**Figure S5.** Changes in the PC, PI, PE and PEp containing fatty acid chains of either arachidonic acid (20:4) or docosahexaenoic acid (22:6). Species marked with asterisk are abundant species in each class of lipids.

Table S1 Relative peak area of lipid species (KO/WT) from cortex, hippocampus and hypothalamus groups obtained by nLC–ESI–MS/MS. The molecular structures of PC, PE, and TAG species are expressed with their total chain length. (N.D.: Not detected, N.Q.: Not quantifiable, p-value: < 0.01 (**) & < 0.05 (*), underlined species are abundant ones in each class of lipids). Numbers (No. 1, 2 .. ) match with individual species in Figure 2. Retention times (t_r_) are based on nLC–ESI–MS/MS analysis of cortex WT sample in negative ion mode for all lipid classes except TAG and PEp in positive ion mode. Retention times of lipid species not detected cortex WT are based on the analysis of other two tissues.

| Class | chain type | m/z | t_r_ | No. | Cortex | | | KO/WT | | Hippocampus | | | | KO/WT | | Hypothalamus | | | KO/WT |
| --- | --- | --- | --- | --- | --- | --- | --- | --- | --- | --- | --- | --- | --- | --- | --- | --- | --- | --- | --- |
|  |  |  | (min) |  | WT | abund. | KO | pooled | individual | WT | abund. | KO | pooled | | individual | WT | abund. | KO | pooled |
| **LPC** | 14:0 | 468.3 | 17.9 |  | 0.00 | 0.2% | 0.01 | 1.06 ± 0.28 | 1.04 ± 0.04 | 0.00 | 0.1% | 0.00 | 1.57 ± 0.10 | | 1.54 ± 0.09** | 0.01 | 0.1% | 0.01 | 1.21 ± 0.09 |
|  | 16:1 | 494.3 | 18.7 |  | 0.03 | 0.9% | 0.03 | 1.01 ± 0.03 | 0.95 ± 0.03 | 0.02 | 0.6% | 0.02 | 0.99 ± 0.11 | | 1.00 ± 0.06 | 0.05 | 1.1% | 0.04 | 0.78 ± 0.08 |
|  | 16:0 | 496.3 | 20.8 | **1** | 1.27 | 43.9% | 1.73 | 1.36 ± 0.08 | 1.51 ± 0.04** | 1.22 | 38.6% | 1.45 | 1.18 ± 0.05 | | 1.17 ± 0.03 | 1.55 | 34.6% | 2.16 | 1.39 ± 0.16 |
|  | 18:2 | 520.3 | 20.4 |  | 0.01 | 0.5% | 0.01 | 0.97 ± 0.03 | 1.07 ± 0.02 | 0.02 | 0.6% | 0.02 | 1.01 ± 0.04 | | 0.96 ± 0.07 | 0.03 | 0.7% | 0.04 | 1.30 ± 0.14 |
|  | 18:1 | 522.3 | 21.2 | **2** | 0.68 | 23.7% | 0.74 | 1.09 ± 0.06 | 1.13 ± 0.04** | 0.77 | 24.4% | 0.91 | 1.18 ± 0.16 | | 1.11 ± 0.05 | 1.62 | 36.0% | 1.83 | 1.13 ± 0.07 |
|  | 18:0 | 524.3 | 22.7 | **3** | 0.60 | 20.8% | 0.57 | 0.95 ± 0.06 | 0.95 ± 0.04 | 0.68 | 21.5% | 0.69 | 1.01 ± 0.08 | | 1.00 ± 0.03 | 0.57 | 12.8% | 0.92 | 1.61 ± 0.12* |
|  | 20:4 | 544.3 | 20.1 |  | 0.12 | 4.0% | 0.15 | 1.26 ± 0.16 | 1.10 ± 0.03 | 0.20 | 6.4% | 0.22 | 1.08 ± 0.05 | | 1.00 ± 0.06 | 0.24 | 5.3% | 0.40 | 1.66 ± 0.09** |
|  | 20:3 | 546.3 | 20.5 |  | 0.01 | 0.4% | 0.01 | 1.24 ± 0.08 | 1.13 ± 0.04** | 0.02 | 0.7% | 0.02 | 0.93 ± 0.12 | | 0.96 ± 0.04 | 0.03 | 0.8% | 0.04 | 1.17 ± 0.09 |
|  | 20:1 | 550.3 | 23.1 |  | 0.03 | 1.0% | 0.03 | 0.96 ± 0.08 | 0.95 ± 0.04 | 0.03 | 1.0% | 0.03 | 0.99 ± 0.14 | | 0.93 ± 0.05 | 0.03 | 0.7% | 0.05 | 1.72 ± 0.21* |
|  | 22:6 | 568.3 | 21.9 |  | 0.10 | 3.6% | 0.08 | 0.75 ± 0.04 | 0.85 ± 0.02** | 0.16 | 4.9% | 0.23 | 1.48 ± 0.06 | | 0.73 ± 0.03 | 0.30 | 6.7% | 0.27 | 0.90 ± 0.04 |
|  | 22:5 | 570.3 | 23.9 |  | 0.01 | 0.3% | 0.01 | 0.92 ± 0.10 | 0.90 ± 0.04 | 0.01 | 0.4% | 0.01 | 0.97 ± 0.22 | | 0.94 ± 0.06 | 0.02 | 0.5% | 0.04 | 1.99 ± 0.21 |
|  | 22:4 | 572.3 | 24.2 |  | 0.02 | 0.5% | 0.02 | 0.98 ± 0.06 | 1.07 ± 0.04 | 0.02 | 0.8% | 0.02 | 0.83 ± 0.13 | | 0.88 ± 0.04 | 0.04 | 0.8% | 0.07 | 2.02 ± 0.13* |
| **PC** | 30:0 | 706.5 | 29.8 |  | 1.01 | 0.8% | 0.71 | 0.70 ± 0.13 | 0.93 ± 0.04 | 0.48 | 0.6% | 0.69 | 1.44 ± 0.19 | | 1.38 ± 0.04** | 1.58 | 1.3% | 1.37 | 0.86 ± 0.09 |
|  | 32:2 | 730.5 | 28.9 |  | 0.73 | 0.6% | 1.00 | 1.36 ± 0.05 | 1.32 ± 0.08** | 0.76 | 0.9% | 1.00 | 1.31 ± 0.12 | | 1.37 ± 0.09** | 0.55 | 0.5% | 0.53 | 0.97 ± 0.07 |
|  | 32:1 | 732.5 | 30.3 | **1** | 7.81 | 6.4% | 7.81 | 1.00 ± 0.11 | 1.03 ± 0.05 | 6.05 | 7.2% | 9.34 | 1.54 ± 0.20 | | 1.48 ± 0.08** | 8.45 | 6.9% | 9.49 | 1.12 ± 0.06 |
|  | 32:0 | 734.5 | 31.8 | **2** | 9.76 | 8.0% | 10.39 | 1.06 ± 0.13 | 1.08 ± 0.07 | 7.33 | 8.7% | 10.59 | 1.44 ± 0.08 | | 1.48 ± 0.05** | 5.57 | 4.6% | 9.81 | 1.76 ± 0.10** |
|  | 34:2 | 758.5 | 30.8 | **3** | 4.00 | 3.3% | 3.39 | 0.85 ± 0.11 | 0.91 ± 0.08 | 3.28 | 3.9% | 3.78 | 1.15 ± 0.13 | | 1.18 ± 0.05** | 3.42 | 2.8% | 4.18 | 1.22 ± 0.06* |
|  | 34:1 | 760.5 | 32.3 | **4** | 12.54 | 10.3% | 13.25 | 1.06 ± 0.09 | 1.03 ± 0.05 | 10.58 | 12.6% | 14.09 | 1.33 ± 0.08 | | 1.28 ± 0.04** | 12.10 | 9.9% | 13.21 | 1.09 ± 0.08 |
|  | 34:0 | 762.5 | 34.2 | **5** | 9.51 | 7.8% | 8.27 | 0.87 ± 0.04 | 0.91 ± 0.07* | 4.69 | 5.6% | 7.87 | 1.68 ± 0.13 | | 1.56 ± 0.07** | 8.01 | 6.5% | 7.61 | 0.95 ± 0.07 |
|  | 36:5 | 780.5 | 29.4 |  | 0.68 | 0.6% | 0.64 | 0.95 ± 0.07 | 0.98 ± 0.07 | 0.73 | 0.9% | 0.98 | 1.34 ± 0.14 | | 1.41 ± 0.09** | 0.44 | 0.4% | 0.53 | 1.21 ± 0.05** |
|  | 36:4 | 782.5 | 30.8 | **6** | 7.92 | 6.5% | 8.31 | 1.05 ± 0.06 | 1.05 ± 0.04 | 5.03 | 6.0% | 6.57 | 1.30 ± 0.14 | | 1.26 ± 0.04** | 9.25 | 7.6% | 9.33 | 1.01 ± 0.04 |
|  | 36:3 | 784.5 | 31.6 | **7** | 4.53 | 3.7% | 4.74 | 1.05 ± 0.07 | 1.11 ± 0.09** | 3.37 | 4.0% | 5.12 | 1.52 ± 0.24 | | 1.39 ± 0.08** | 5.69 | 4.7% | 3.34 | 0.59 ± 0.04** |
|  | 36:2 | 786.5 | 32.7 | **8** | 3.16 | 2.6% | 3.38 | 1.07 ± 0.05 | 1.12 ± 0.04* | 3.43 | 4.1% | 4.85 | 1.41 ± 0.12 | | 1.37 ± 0.06** | 3.91 | 3.2% | 3.15 | 0.81 ± 0.04* |
|  | 36:1 | 788.5 | 34.5 | **9** | 9.47 | 7.8% | 7.90 | 0.83 ± 0.02 | 0.85 ± 0.03** | 5.53 | 6.6% | 9.43 | 1.70 ± 0.19 | | 1.74 ± 0.06** | 8.57 | 7.0% | 10.27 | 1.20 ± 0.06 |
|  | 36:0 | 790.5 | 35.0 |  | 2.55 | 2.1% | 2.12 | 0.83 ± 0.13 | 0.85 ± 0.02** | 1.78 | 2.1% | 2.60 | 1.46 ± 0.12 | | 1.41 ± 0.05** | 2.63 | 2.1% | 2.40 | 0.91 ± 0.07 |
|  | 38:7 | 804.5 | 28.8 |  | 0.66 | 0.5% | 0.66 | 1.00 ± 0.04 | 1.00 ± 0.03 | 0.64 | 0.8% | 0.75 | 1.18 ± 0.05 | | 1.21 ± 0.06** | 0.32 | 0.3% | 0.39 | 1.25 ± 0.09* |
|  | 38:6 | 806.5 | 30.3 | **10** | 7.46 | 6.2% | 7.84 | 1.05 ± 0.09 | 1.08 ± 0.05** | 4.73 | 5.6% | 6.63 | 1.40 ± 0.09 | | 1.45 ± 0.06** | 10.45 | 8.5% | 8.28 | 0.79 ± 0.04 |
|  | 38:5 | 808.5 | 30.8 | **11** | 5.98 | 4.9% | 6.05 | 1.01 ± 0.18 | 1.14 ± 0.04** | 4.52 | 5.4% | 7.26 | 1.61 ± 0.15 | | 1.50 ± 0.06** | 5.92 | 4.8% | 7.39 | 1.25 ± 0.07** |
|  | 38:4 | 810.5 | 33.2 | **12** | 5.34 | 4.4% | 5.53 | 1.03 ± 0.04 | 1.08 ± 0.05** | 5.30 | 6.3% | 8.12 | 1.53 ± 0.20 | | 1.47 ± 0.06** | 4.58 | 3.7% | 5.80 | 1.27 ± 0.08* |
|  | 38:3 | 812.5 | 34.0 |  | 1.34 | 1.1% | 1.24 | 0.93 ± 0.03 | 1.01 ± 0.05 | 1.65 | 2.0% | 2.13 | 1.29 ± 0.13 | | 1.28 ± 0.08** | 1.15 | 0.9% | 1.40 | 1.23 ± 0.10 |
|  | 38:2 | 814.5 | 35.2 | **13** | 3.27 | 2.7% | 2.47 | 0.76 ± 0.08 | 0.82 ± 0.07** | 1.72 | 2.0% | 2.52 | 1.47 ± 0.31 | | 1.37 ± 0.13** | 3.48 | 2.8% | 2.92 | 0.84 ± 0.05 |
|  | 38:1 | 816.5 | 36.8 | **14** | 4.42 | 3.6% | 1.51 | 0.34 ± 0.03 | 0.33 ± 0.02** | 1.02 | 1.2% | 1.16 | 1.14 ± 0.11 | | 1.25 ± 0.07* | 5.81 | 4.7% | 4.09 | 0.71 ± 0.07 |
|  | 38:0 | 818.5 | 38.6 |  | 0.71 | 0.6% | 0.23 | 0.33 ± 0.01 | 0.36 ± 0.02** | 0.26 | 0.3% | 0.35 | 1.32 ± 0.28 | | 1.42 ± 0.07** | 0.59 | 0.5% | 0.58 | 0.98 ± 0.09 |
|  | 40:8 | 830.5 | 29.8 |  | 1.01 | 0.8% | 0.77 | 0.76 ± 0.06 | 0.74 ± 0.05** | 0.35 | 0.4% | 0.52 | 1.48 ± 0.19 | | 1.54 ± 0.06** | 0.91 | 0.7% | 0.75 | 0.83 ± 0.05* |
|  | 40:7 | 832.5 | 30.8 | **15** | 3.16 | 2.6% | 3.32 | 1.05 ± 0.05 | 1.08 ± 0.06 | 2.04 | 2.4% | 3.07 | 1.50 ± 0.21 | | 1.45 ± 0.08** | 3.95 | 3.2% | 2.71 | 0.68 ± 0.03** |
|  | 40:6 | 834.5 | 32.9 | **16** | 5.23 | 4.3% | 5.36 | 1.02 ± 0.02 | 1.00 ± 0.04 | 3.00 | 3.6% | 4.62 | 1.54 ± 0.14 | | 1.47 ± 0.06** | 5.08 | 4.2% | 6.74 | 1.32 ± 0.10 |
|  | 40:5 | 836.5 | 33.3 |  | 1.26 | 1.0% | 1.17 | 0.93 ± 0.09 | 1.00 ± 0.04 | 1.48 | 1.8% | 2.36 | 1.60 ± 0.30 | | 1.54 ± 0.10** | 1.04 | 0.9% | 1.01 | 0.97 ± 0.10 |
|  | 40:4 | 838.5 | 34.4 |  | 1.59 | 1.3% | 1.60 | 1.01 ± 0.23 | 0.97 ± 0.08 | 1.44 | 1.7% | 1.89 | 1.31 ± 0.24 | | 1.55 ± 0.09** | 2.55 | 2.1% | 1.79 | 0.70 ± 0.05* |
|  | 40:3 | 840.5 | 36.3 |  | 0.10 | 0.1% | 0.10 | 1.02 ± 0.13 | 0.94 ± 0.04 | 0.20 | 0.2% | 0.31 | 1.59 ± 0.05 | | 1.49 ± 0.06** | 0.08 | 0.1% | 0.10 | 1.30 ± 0.10* |
|  | 40:2 | 842.5 | 37.1 |  | 0.82 | 0.7% | 0.35 | 0.43 ± 0.02 | 0.42 ± 0.03** | 0.13 | 0.2% | 0.23 | 1.16 ± 0.21 | | 1.12 ± 0.05 | 1.05 | 0.9% | 0.99 | 0.94 ± 0.07 |
|  | 40:1 | 844.5 | 39.2 |  | 1.46 | 1.2% | 0.35 | 0.24 ± 0.01 | 0.21 ± 0.01** | 0.20 | 0.2% | 0.36 | 1.76 ± 0.12 | | 1.65 ± 0.07** | 1.75 | 1.4% | 1.05 | 0.60 ± 0.09* |
|  | 40:0 | 846.5 | 41.1 |  | 0.31 | 0.3% | 0.10 | 0.34 ± 0.01 | 0.31 ± 0.01** | 0.05 | 0.1% | 0.11 | 2.02 ± 0.39 | | 2.00 ± 0.21** | 0.32 | 0.3% | 0.19 | 0.61 ± 0.04* |
|  | 42:10 | 854.5 | 29.1 |  | 0.92 | 0.8% | 1.02 | 1.10 ± 0.13 | 1.06 ± 0.05 | 1.10 | 1.3% | 1.20 | 1.09 ± 0.08 | | 1.09 ± 0.09 | 0.57 | 0.5% | 0.71 | 1.26 ± 0.08 |
|  | 42:8 | 858.5 | 31.5 |  | 0.17 | 0.1% | 0.17 | 0.95 ± 0.05 | 0.91 ± 0.03** | 0.15 | 0.2% | 0.22 | 1.44 ± 0.19 | | 1.41 ± 0.05** | 0.24 | 0.2% | 0.19 | 0.80 ± 0.05 |
|  | 42:2 | 870.5 | 39.4 |  | 0.67 | 0.5% | 0.22 | 0.33 ± 0.07 | 0.31 ± 0.01** | 0.08 | 0.1% | 0.16 | 1.92 ± 0.26 | | 2.07 ± 0.16** | 0.86 | 0.7% | 0.74 | 0.86 ± 0.09 |
|  | 42:1 | 872.5 | 41.3 |  | 0.75 | 0.6% | 0.36 | 0.48 ± 0.03 | 0.42 ± 0.03** | 0.20 | 0.2% | 0.33 | 1.65 ± 0.17 | | 1.73 ± 0.17** | 0.86 | 0.7% | 0.58 | 0.67 ± 0.09* |
|  | 44:12 | 878.5 | 31.5 |  | 0.83 | 0.7% | 0.87 | 1.04 ± 0.09 | 1.14 ± 0.07 | 0.82 | 1.0% | 0.93 | 1.14 ± 0.08 | | 1.21 ± 0.12 | 0.53 | 0.4% | 0.61 | 1.15 ± 0.05* |
| **LPE** | 16:1 | 452.3 | 16.6 |  | N.D. | N/A | N.D. | N.D. | N.D. | 0.01 | 0.2% | 0.01 | 0.76 ± 0.17 | | 0.83 ± 0.03** | 0.02 | 0.3% | 0.04 | 2.48 ± 0.27* |
|  | 16:0 | 454.3 | 20.3 |  | 0.36 | 5.5% | 0.33 | 0.92 ± 0.03 | 0.89 ± 0.02** | 0.34 | 4.7% | 0.35 | 1.01 ± 0.04 | | 0.99 ± 0.04 | 0.32 | 5.0% | 0.42 | 1.31 ± 0.17 |
|  | 18:2 | 478.3 | 19.2 |  | N.Q. | N/A | N.Q. | N.Q. | N.Q. | N.Q. | N/A | N.Q. | N.Q. | | N.Q. | N.D. | N/A | N.D. | N.D. |
|  | 18:1 | 480.3 | 20.7 | **1** | 0.98 | 15.1% | 0.83 | 0.85 ± 0.03 | 0.86 ± 0.02** | 1.14 | 15.7% | 1.12 | 0.98 ± 0.06 | | 1.12 ± 0.04** | 1.22 | 19.1% | 1.77 | 1.45 ± 0.11** |
|  | 18:0 | 482.3 | 22.7 | **2** | 2.07 | 31.9% | 1.88 | 0.91 ± 0.04 | 0.96 ± 0.03 | 2.56 | 35.3% | 2.24 | 0.88 ± 0.16 | | 1.00 ± 0.05 | 1.63 | 25.4% | 2.70 | 1.66 ± 0.15** |
|  | 20:5 | 500.3 | 16.2 |  | N.D. | N/A | N.D. | N.D. | N.D. | N.Q. | N/A | N.Q. | N.Q. | | N.Q. | N.D. | N/A | N.D. | N.D. |
|  | 20:4 | 502.3 | 19.5 |  | 0.34 | 5.3% | 0.34 | 1.00 ± 0.04 | 1.01 ± 0.02 | 0.34 | 4.7% | 0.32 | 0.94 ± 0.08 | | 0.99 ± 0.04 | 0.45 | 7.0% | 0.48 | 1.07 ± 0.08 |
|  | 20:3 | 504.3 | 20.2 |  | N.Q. | N/A | N.Q. | N.Q. | N.Q. | 0.06 | 0.8% | 0.06 | 0.94 ± 0.06 | | 0.91 ± 0.02* | 0.06 | 1.0% | 0.07 | 1.06 ± 0.04 |
|  | 20:2 | 506.3 | 21.3 |  | N.D. | N/A | N.D. | N.D. | N.D. | 0.05 | 0.7% | 0.05 | 1.03 ± 0.09 | | 0.92 ± 0.04** | 0.05 | 0.8% | 0.07 | 1.47 ± 0.14* |
|  | 20:1 | 508.3 | 22.5 | **3** | 0.58 | 9.0% | 0.66 | 1.14 ± 0.09 | 1.09 ± 0.03 | 0.93 | 12.8% | 0.68 | 0.73 ± 0.10 | | 0.87 ± 0.06** | 0.90 | 14.1% | 1.07 | 1.18 ± 0.18 |
|  | 22:6 | 526.3 | 19.5 | **4** | 1.36 | 21.0% | 1.35 | 1.00 ± 0.07 | 1.05 ± 0.04 | 0.95 | 13.2% | 1.00 | 1.04 ± 0.15 | | 1.04 ± 0.04 | 0.58 | 9.0% | 1.79 | 3.10 ± 0.36* |
|  | 22:5 | 528.3 | 19.7 |  | 0.10 | 1.5% | 0.07 | 0.69 ± 0.03 | 0.65 ± 0.02** | 0.07 | 1.0% | 0.08 | 1.11 ± 0.18 | | 1.03 ± 0.04 | 0.13 | 2.1% | 0.14 | 1.08 ± 0.08 |
|  | 22:4 | 530.3 | 20.9 | **5** | 0.35 | 5.4% | 0.38 | 1.07 ± 0.05 | 0.97 ± 0.03 | 0.54 | 7.4% | 0.50 | 0.94 ± 0.07 | | 0.93 ± 0.04 | 0.58 | 9.1% | 0.72 | 1.24 ± 0.12 |
|  | 24:6 | 554.3 | 21.2 |  | 0.28 | 4.4% | 0.16 | 0.55 ± 0.05 | 0.54 ± 0.01** | 0.13 | 1.8% | 0.10 | 0.76 ± 0.06 | | 0.80 ± 0.04** | 0.30 | 4.6% | 0.41 | 1.39 ± 0.02** |
|  | 24:4 | 558.3 | 22.6 |  | 0.06 | 0.9% | 0.08 | 1.40 ± 0.07 | 1.44 ± 0.06** | 0.10 | 1.4% | 0.10 | 0.92 ± 0.03 | | 1.07 ± 0.07 | 0.17 | 2.7% | 0.19 | 1.09 ± 0.09 |
| **PE** | 32:1 | 690.5 | 29.5 |  | 0.47 | 0.2% | 0.67 | 1.40 ± 0.06 | 1.44 ± 0.05** | 0.67 | 0.2% | 0.67 | 1.00 ± 0.11 | | 1.00 ± 0.05 | 1.13 | 0.3% | 0.83 | 0.73 ± 0.09 |
|  | 34:2 | 716.5 | 31.6 |  | 1.47 | 0.5% | 2.08 | 1.42 ± 0.15 | 1.49 ± 0.04** | 2.63 | 0.6% | 2.26 | 0.86 ± 0.19 | | 0.88 ± 0.06 | 4.05 | 1.0% | 3.61 | 0.89 ± 0.11 |
|  | 34:1 | 718.5 | 32.3 |  | 12.67 | 4.1% | 11.03 | 0.87 ± 0.03 | 0.93 ± 0.03** | 11.32 | 2.8% | 10.92 | 0.96 ± 0.05 | | 1.00 ± 0.02 | 10.97 | 2.8% | 10.45 | 0.95 ± 0.09 |
|  | 34:0 | 720.5 | 33.2 |  | 3.53 | 1.1% | 2.46 | 0.70 ± 0.11 | 0.66 ± 0.03** | 2.57 | 0.6% | 1.78 | 0.69 ± 0.07 | | 0.73 ± 0.07* | 2.91 | 0.8% | 2.62 | 0.90 ± 0.11 |
|  | 36:5 | 738.5 | 28.1 |  | 1.02 | 0.3% | 1.44 | 1.41 ± 0.25 | 1.53 ± 0.05** | 1.53 | 0.4% | 1.64 | 1.07 ± 0.14 | | 1.05 ± 0.07 | 1.51 | 0.4% | 0.52 | 0.34 ± 0.03* |
|  | 36:4 | 740.5 | 30.0 | **1** | 11.71 | 3.8% | 19.33 | 1.65 ± 0.24 | 1.75 ± 0.08** | 25.83 | 6.4% | 22.19 | 0.86 ± 0.11 | | 0.88 ± 0.06 | 24.87 | 6.4% | 34.06 | 1.37 ± 0.11* |
|  | 36:3 | 742.5 | 30.3 |  | 2.03 | 0.7% | 3.74 | 1.84 ± 0.14 | 1.76 ± 0.06 | 5.00 | 1.2% | 3.95 | 0.79 ± 0.13 | | 0.72 ± 0.05** | 4.76 | 1.2% | 5.92 | 1.24 ± 0.09* |
|  | 36:2 | 744.5 | 32.5 |  | 13.56 | 4.4% | 13.39 | 0.99 ± 0.12 | 0.99 ± 0.03** | 13.43 | 3.3% | 11.39 | 0.85 ± 0.09 | | 0.91 ± 0.04 | 15.41 | 4.0% | 10.37 | 0.67 ± 0.04** |
|  | 36:1 | 746.5 | 34.2 | **2** | 16.73 | 5.4% | 20.61 | 1.23 ± 0.08 | 1.16 ± 0.04** | 21.75 | 5.4% | 24.18 | 1.11 ± 0.11 | | 0.96 ± 0.04 | 15.21 | 3.9% | 9.51 | 0.62 ± 0.08 |
|  | 38:6 | 764.5 | 29.8 | **3** | 51.70 | 16.7% | 84.48 | 1.63 ± 0.46 | 1.64 ± 0.08** | 76.81 | 19.0% | 64.97 | 0.84 ± 0.07 | | 0.86 ± 0.05** | 84.62 | 21.8% | 29.17 | 0.34 ± 0.03* |
|  | 38:5 | 766.5 | 30.3 | **4** | 20.44 | 6.6% | 35.22 | 1.72 ± 0.24 | 1.71 ± 0.07** | 46.52 | 11.5% | 46.45 | 1.00 ± 0.16 | | 0.86 ± 0.04 | 47.44 | 12.2% | 27.12 | 0.57 ± 0.09 |
|  | 38:4 | 768.5 | 32.7 | **5** | 54.10 | 17.4% | 58.16 | 1.07 ± 0.04 | 1.11 ± 0.04 | 71.71 | 17.7% | 68.45 | 0.95 ± 0.14 | | 0.92 ± 0.04 | 39.38 | 10.2% | 42.71 | 1.08 ± 0.06 |
|  | 38:3 | 770.5 | 33.4 |  | 10.21 | 3.3% | 9.57 | 0.94 ± 0.05 | 1.00 ± 0.03 | 11.21 | 2.8% | 9.99 | 0.89 ± 0.09 | | 0.94 ± 0.05 | 9.35 | 2.4% | 7.36 | 0.79 ± 0.03 |
|  | 38:2 | 772.5 | 34.4 |  | 2.00 | 0.6% | 1.98 | 0.99 ± 0.05 | 0.99 ± 0.03 | 2.23 | 0.6% | 2.06 | 0.92 ± 0.11 | | 1.00 ± 0.04 | 2.31 | 0.6% | 1.18 | 0.51 ± 0.07* |
|  | 38:1 | 774.5 | 36.2 |  | 3.05 | 1.0% | 3.26 | 1.07 ± 0.12 | 1.08 ± 0.05 ** | 0.98 | 0.2% | 0.74 | 0.75 ± 0.14 | | 1.03 ± 0.11 | 5.82 | 1.5% | 2.17 | 0.37 ± 0.04* |
|  | 40:7 | 790.5 | 29.5 | **6** | 19.51 | 6.3% | 29.73 | 1.52 ± 0.29 | 1.60 ± 0.08 | 23.13 | 5.7% | 19.75 | 0.85 ± 0.05 | | 0.97 ± 0.05 | 27.54 | 7.1% | 17.79 | 0.65 ± 0.05** |
|  | 40:6 | 792.5 | 32.4 | **7** | 62.57 | 20.2% | 68.88 | 1.10 ± 0.07 | 1.04 ± 0.05** | 57.05 | 14.1% | 56.30 | 0.99 ± 0.10 | | 1.02 ± 0.03 | 59.93 | 15.5% | 29.81 | 0.50 ± 0.02* |
|  | 40:5 | 794.5 | 33.5 |  | 8.08 | 2.6% | 10.29 | 1.27 ± 0.12 | 1.24 ± 0.04* | 10.49 | 2.6% | 10.68 | 1.02 ± 0.07 | | 1.02 ± 0.02 | 7.10 | 1.8% | 7.09 | 1.00 ± 0.16 |
|  | 40:4 | 796.5 | 34.2 |  | 9.63 | 3.1% | 10.12 | 1.05 ± 0.08 | 1.09 ± 0.04 | 11.42 | 2.8% | 10.27 | 0.90 ± 0.18 | | 1.02 ± 0.08 | 10.73 | 2.8% | 10.84 | 1.01 ± 0.06 |
|  | 40:3 | 798.5 | 35.6 |  | 1.44 | 0.5% | 1.58 | 1.10 ± 0.06 | 1.02 ± 0.02** | 1.52 | 0.4% | 1.99 | 1.31 ± 0.12 | | 1.18 ± 0.07* | 2.03 | 0.5% | 1.05 | 0.52 ± 0.05** |
|  | 42:6 | 820.5 | 34.9 |  | 0.25 | 0.1% | 0.32 | 1.28 ± 0.08 | 1.23 ± 0.03 | 0.27 | 0.1% | 0.24 | 0.89 ± 0.05 | | 0.89 ± 0.05 | 0.34 | 0.1% | 0.38 | 1.12 ± 0.23 |
|  | 42:4 | 824.5 | 36.9 |  | 0.51 | 0.2% | 0.48 | 0.95 ± 0.09 | 0.96 ± 0.03** | 0.53 | 0.1% | 0.33 | 0.62 ± 0.20 | | 0.60 ± 0.04** | 0.44 | 0.1% | 0.35 | 0.80 ± 0.09 |
|  | 44:10 | 840.5 | 31.8 |  | 3.53 | 1.1% | 5.31 | 1.50 ± 0.15 | 1.65 ± 0.06 | 6.68 | 1.6% | 6.54 | 0.98 ± 0.08 | | 1.14 ± 0.10 | 9.90 | 2.6% | 9.06 | 0.91 ± 0.08 |
| **PEp** | 16:0p/18:1 | 702.6 | 37.8 | **1** | 6.53 | 4.0% | 5.63 | 0.86 ± 0.07 | 0.86 ± 0.03** | 6.27 | 2.5% | 5.05 | 0.80 ± 0.11 | | 0.85 ± 0.05 | 4.18 | 2.1% | 2.87 | 0.69 ± 0.02** |
|  | 18:1p/16:0 | 702.6 | 37.6 |  | 2.57 | 1.6% | 2.94 | 1.15 ± 0.15 | 1.15 ± 0.04** | 3.55 | 1.4% | 3.51 | 0.99 ± 0.13 | | 1.01 ± 0.06 | 1.85 | 0.9% | 0.99 | 0.54 ± 0.03** |
|  | 18:0p/16:1 | 702.6 | 37.6 |  | 2.55 | 1.5% | 3.41 | 1.34 ± 0.18 | 1.55 ± 0.06** | 4.89 | 2.0% | 3.17 | 0.65 ± 0.15 | | 0.72 ± 0.06** | 1.31 | 0.6% | 0.99 | 0.75 ± 0.08 |
|  | 16:0p/20:4 | 724.6 | 35.7 |  | 5.47 | 3.3% | 3.45 | 0.63 ± 0.03 | 0.73 ± 0.04** | 5.28 | 2.1% | 5.11 | 0.97 ± 0.25 | | 1.00 ± 0.06 | 4.90 | 2.4% | 1.90 | 0.39 ± 0.04** |
|  | 18:1p/18:1 | 728.6 | 38.2 | **2** | 17.07 | 10.4% | 18.90 | 1.11 ± 0.10 | 1.33 ± 0.05** | 23.76 | 9.6% | 21.28 | 0.90 ± 0.15 | | 0.90 ± 0.05 | 14.11 | 7.0% | 9.78 | 0.69 ± 0.06* |
|  | 16:0p/20:1 | 730.6 | 40.0 |  | 2.83 | 1.7% | 3.92 | 1.38 ± 0.19 | 1.29 ± 0.04** | 1.86 | 0.8% | 1.67 | 0.90 ± 0.15 | | 0.93 ± 0.05 | 2.24 | 1.1% | 1.82 | 0.81 ± 0.05 |
|  | 18:0p/18:1 | 730.6 | 40.0 | **3** | 11.75 | 7.1% | 12.73 | 1.08 ± 0.22 | 1.20 ± 0.05** | 9.65 | 3.9% | 9.46 | 0.98 ± 0.06 | | 0.96 ± 0.05 | 6.87 | 3.4% | 4.89 | 0.71 ± 0.03 |
|  | 18:1p/18:0 | 730.6 | 39.4 |  | 1.01 | 0.6% | 1.34 | 1.33 ± 0.04 | 1.32 ± 0.03** | 1.19 | 0.5% | 1.05 | 0.88 ± 0.08 | | 0.89 ± 0.06 | 1.11 | 0.5% | 0.67 | 0.60 ± 0.06* |
|  | 18:0p/18:0 | 732.6 | 39.8 |  | 0.44 | 0.3% | 0.50 | 1.12 ± 0.07 | 1.20 ± 0.03** | 0.37 | 0.2% | 0.37 | 0.99 ± 0.10 | | 0.95 ± 0.04 | 0.29 | 0.1% | 0.25 | 0.87 ± 0.09 |
|  | 16:1p/22:6 | 746.6 | 33.1 |  | N.D. | N/A | N.D. | N.D. | N.D. | 0.17 | 0.1% | 0.13 | 0.81 ± 0.20 | | 0.86 ± 0.03 | N.D. | N/A | N.D. | N.D. |
|  | 16:0p/22:6 | 748.6 | 35.4 | **4** | 20.43 | 12.4% | 39.29 | 1.92 ± 0.21 | 2.02 ± 0.08** | 41.88 | 17.0% | 34.69 | 0.83 ± 0.11 | | 0.83 ± 0.04 | 17.34 | 8.6% | 17.70 | 1.02 ± 0.10 |
|  | 16:0p/22:5 | 750.6 | 35.9 |  | 1.57 | 1.0% | 2.66 | 1.69 ± 0.12 | 1.69 ± 0.04** | 3.20 | 1.3% | 2.51 | 0.79 ± 0.08 | | 0.68 ± 0.04** | 2.27 | 1.1% | 1.65 | 0.73 ± 0.04** |
|  | 18:1p/20:4 | 750.6 | 35.8 | **5** | 10.48 | 6.4% | 9.99 | 0.95 ± 0.08 | 0.99 ± 0.02** | 14.73 | 6.0% | 9.66 | 0.66 ± 0.09 | | 0.67 ± 0.05 | 14.13 | 7.0% | 6.65 | 0.47 ± 0.03** |
|  | 16:0p/22:4 | 752.6 | 37.5 | **6** | 4.14 | 2.5% | 5.60 | 1.35 ± 0.08 | 1.38 ± 0.05** | 6.80 | 2.8% | 10.01 | 1.47 ± 0.17 | | 1.27 ± 0.06** | 4.65 | 2.3% | 6.80 | 1.46 ± 0.09** |
|  | 16:1p/22:3 | 752.6 | 35.8 | **7** | 5.93 | 3.6% | 6.12 | 1.03 ± 0.03 | 1.22 ± 0.06** | 7.70 | 3.1% | 6.29 | 0.82 ± 0.07 | | 0.95 ± 0.07 | 6.72 | 3.3% | 4.61 | 0.69 ± 0.02** |
|  | 18:0p/20:4 | 752.6 | 38.4 | **8** | 13.20 | 8.0% | 16.66 | 1.26 ± 0.08 | 1.39 ± 0.04 | 19.04 | 7.7% | 18.03 | 0.95 ± 0.09 | | 1.05 ± 0.05 | 16.76 | 8.3% | 11.97 | 0.71 ± 0.07* |
|  | 16:1p/22:2 | 754.6 | 41.0 |  | 0.96 | 0.6% | 1.03 | 1.07 ± 0.14 | 1.08 ± 0.05* | 1.23 | 0.5% | 1.06 | 0.87 ± 0.14 | | 0.95 ± 0.06 | 0.95 | 0.5% | 0.90 | 0.96 ± 0.10 |
|  | 18:0p/20:3 | 754.6 | 39.7 |  | 1.30 | 0.8% | 1.42 | 1.10 ± 0.15 | 1.22 ± 0.05* | 1.97 | 0.8% | 2.00 | 1.02 ± 0.10 | | 1.02 ± 0.04 | 1.85 | 0.9% | 1.67 | 0.90 ± 0.13 |
|  | 18:1p/20:1 | 756.6 | 40.0 | **9** | 5.08 | 3.1% | 10.19 | 2.01 ± 0.16 | 1.96 ± 0.05** | 7.21 | 2.9% | 6.83 | 0.95 ± 0.12 | | 0.89 ± 0.07 | 12.96 | 6.4% | 4.67 | 0.36 ± 0.03* |
|  | 18:0p/20:2 | 756.6 | 40.7 |  | 0.25 | 0.1% | 0.34 | 1.37 ± 0.12 | 1.40 ± 0.05** | 0.33 | 0.1% | 0.24 | 0.72 ± 0.14 | | 1.00 ± 0.06 | 0.39 | 0.2% | 0.30 | 0.76 ± 0.08 |
|  | 20:1p/18:1 | 756.6 | 40.7 |  | N.D. | N/A | N.D. | N.D. | N.D. | 0.24 | 0.1% | 0.26 | 1.05 ± 0.15 | | 0.98 ± 0.06 | 0.25 | 0.1% | 0.14 | 0.57 ± 0.11** |
|  | 18:0p/20:1 | 758.6 | 43.1 | **10** | 4.20 | 2.6% | 3.17 | 0.76 ± 0.08 | 0.73 ± 0.02** | 1.80 | 0.7% | 1.65 | 0.92 ± 0.04 | | 0.99 ± 0.04 | 7.27 | 3.6% | 3.25 | 0.45 ± 0.05** |
|  | 20:0p/18:1 | 758.6 | 46.8 |  | N.D. | N/A | N.D. | N.D. | N.D. | N.D. | N/A | N.D. | N.D. | | N.D. | 0.39 | 0.2% | 0.33 | 0.83 ± 0.06* |
|  | 18:0p/20:0 | 760.6 | 43.9 |  | N.Q. | N/A | N.Q. | N.Q. | N.Q. | 0.37 | 0.2% | 0.31 | 0.83 ± 0.10 | | 0.92 ± 0.04 | 0.66 | 0.3% | 0.22 | 0.33 ± 0.07 |
|  | 18:1p/22:6 | 774.6 | 36.3 | **11** | 9.76 | 5.9% | 19.15 | 1.96 ± 0.23 | 2.05 ± 0.07** | 20.86 | 8.5% | 18.53 | 0.89 ± 0.12 | | 1.00 ± 0.07 | 11.34 | 5.6% | 5.58 | 0.49 ± 0.02** |
|  | 18:0p/22:6 | 776.6 | 36.9 | **12** | 21.84 | 13.3% | 32.56 | 1.49 ± 0.11 | 1.53 ± 0.04** | 33.62 | 13.6% | 26.06 | 0.78 ± 0.07 | | 0.80 ± 0.04** | 41.07 | 20.2% | 23.14 | 0.56 ± 0.05* |
|  | 18:1p/22:5 | 776.6 | 38.0 |  | 1.05 | 0.6% | 1.49 | 1.42 ± 0.06 | 1.55 ± 0.06** | 1.93 | 0.8% | 2.11 | 1.09 ± 0.28 | | 1.13 ± 0.09 | 1.46 | 0.7% | 0.75 | 0.51 ± 0.03* |
|  | 18:0p/22:5 | 778.6 | 38.4 |  | 2.37 | 1.4% | 2.51 | 1.06 ± 0.07 | 1.16 ± 0.03** | 2.47 | 1.0% | 2.37 | 0.96 ± 0.15 | | 0.91 ± 0.04 | 3.51 | 1.7% | 2.08 | 0.59 ± 0.07* |
|  | 18:1p/22:4 | 778.6 | 39.0 | **13** | 5.31 | 3.2% | 7.71 | 1.45 ± 0.08 | 1.47 ± 0.04** | 8.55 | 3.5% | 7.05 | 0.82 ± 0.07 | | 0.84 ± 0.04** | 11.73 | 5.8% | 6.87 | 0.59 ± 0.04* |
|  | 16:0p/24:4 | 780.6 | 39.1 |  | N.D. | N/A | N.D. | N.D. | N.D. | 2.50 | 1.0% | 2.49 | 1.00 ± 0.12 | | 0.98 ± 0.03 | 0.19 | 0.1% | 0.07 | 0.37 ± 0.04** |
|  | 18:0p/22:4 | 780.6 | 39.7 | **14** | 5.11 | 3.1% | 9.44 | 1.85 ± 0.10 | 1.92 ± 0.05** | 11.46 | 4.6% | 8.79 | 0.77 ± 0.06 | | 0.78 ± 0.04** | 6.14 | 3.0% | 3.96 | 0.64 ± 0.10* |
|  | 20:0p/20:4 | 780.6 | 40.0 |  | 0.26 | 0.2% | 0.35 | 1.32 ± 0.18 | 1.48 ± 0.04** | 0.28 | 0.1% | 0.28 | 1.00 ± 0.17 | | 0.98 ± 0.06 | 0.30 | 0.1% | 0.20 | 0.69 ± 0.05* |
|  | 18:1p/22:1 | 784.6 | 41.8 |  | 0.63 | 0.4% | 0.28 | 0.45 ± 0.02 | 0.45 ± 0.02** | 0.30 | 0.1% | 0.25 | 0.83 ± 0.11 | | 1.01 ± 0.06 | 0.69 | 0.3% | 0.32 | 0.46 ± 0.05* |
|  | 18:0p/22:1 | 786.6 | 44.1 |  | 0.54 | 0.3% | 0.27 | 0.50 ± 0.04 | 0.51 ± 0.03** | N.D. | N/A | N.D. | N.D. | | N.D. | 0.98 | 0.5% | 0.48 | 0.49 ± 0.06** |
|  | 20:0p/20:1 | 786.6 | 44.1 |  | N.Q. | N/A | N.Q. | N.Q. | N.Q. | 0.08 | 0.0% | 0.08 | 1.02 ± 0.11 | | 1.14 ± 0.08 | 0.40 | 0.2% | 0.10 | 0.26 ± 0.02** |
|  | 18:0p/24:6 | 804.6 | 39.9 |  | N.D. | N/A | N.D. | N.D. | N.D. | N.D. | N/A | N.D. | N.D. | | N.D. | N.Q. | N/A | N.Q. | N.Q. |
|  | 20:0p/22:6 | 804.6 | 39.9 |  | N.Q. | N/A | N.Q. | N.Q. | N.Q. | 0.27 | 0.1% | 0.29 | 1.06 ± 0.15 | | 1.24 ± 0.09 | 0.24 | 0.1% | 0.29 | 1.23 ± 0.11 |
|  | 18:0p/24:5 | 806.6 | 40.0 |  | N.Q. | N/A | N.Q. | N.Q. | N.Q. | 0.17 | 0.1% | 0.15 | 0.90 ± 0.17 | | 0.93 ± 0.04 | 0.41 | 0.2% | 0.11 | 0.27 ± 0.03** |
|  | 18:1p/24:4 | 806.6 | 39.4 |  | N.Q. | N/A | N.Q. | N.Q. | N.Q. | 0.20 | 0.1% | 0.19 | 0.95 ± 0.05 | | 0.90 ± 0.04** | 0.21 | 0.1% | 0.06 | 0.27 ± 0.02* |
|  | 20:1p/22:4 | 806.6 | 40.0 |  | N.Q. | N/A | N.Q. | N.Q. | N.Q. | 0.17 | 0.1% | 0.17 | 0.99 ± 0.13 | | 1.11 ± 0.10 | 0.24 | 0.1% | 0.10 | 0.42 ± 0.06 |
|  | 18:0p/24:4 | 808.6 | 41.7 |  | N.D. | N/A | N.D. | N.D. | N.D. | N.D. | N/A | N.D. | N.D. | | N.D. | 0.27 | 0.1% | 0.32 | 1.16 ± 0.20 |
|  | 20:0p/22:4 | 808.6 | 42.0 |  | N.D. | N/A | N.D. | N.D. | N.D. | 0.07 | 0.0% | 0.06 | 0.80 ± 0.14 | | 1.12 ± 0.09 | N.D. | N/A | N.D. | N.D. |
|  | 18:1p/24:1 | 812.6 | 45.1 |  | N.D. | N/A | N.D. | N.D. | N.D. | 0.04 | 0.0% | 0.03 | 0.83 ± 0.21 | | 1.12 ± 0.06 | 0.24 | 0.1% | 0.16 | 0.68 ± 0.11 |
| **LPG** | 14:0 | 455.5 | 13.9 | **1** | 0.00 | 1.6% | 0.00 | 2.38 ± 0.62 | 1.73 ± 0.31 | 0.04 | 43.4% | 0.00 | 0.05 ± 0.01 | | 0.06 ± 0.01** | 0.00 | 3.9% | 0.00 | 0.89 ± 0.10 |
|  | 16:1 | 481.5 | 15.3 |  | 0.00 | 4.0% | 0.00 | 0.90 ± 0.11 | 0.78 ± 0.08** | 0.00 | 1.8% | 0.00 | 1.05 ± 0.12 | | 0.95 ± 0.07 | 0.01 | 7.1% | 0.00 | 0.66 ± 0.08 |
|  | 16:0 | 483.5 | 17.5 |  | 0.01 | 11.4% | 0.01 | 1.28 ± 0.13 | 1.38 ± 0.06** | 0.01 | 6.9% | 0.01 | 1.05 ± 0.15 | | 0.99 ± 0.04 | 0.01 | 9.9% | 0.01 | 1.00 ± 0.06 |
|  | 18:1 | 509.5 | 18.0 | **2** | 0.03 | 42.4% | 0.03 | 0.89 ± 0.03 | 0.93 ± 0.02** | 0.02 | 24.9% | 0.02 | 0.93 ± 0.08 | | 0.88 ± 0.03 | 0.03 | 37.2% | 0.02 | 0.71 ± 0.05** |
|  | 18:0 | 511.5 | 20.6 | **3** | 0.03 | 38.2% | 0.02 | 0.83 ± 0.04 | 0.86 ± 0.02** | 0.02 | 19.5% | 0.01 | 0.77 ± 0.12 | | 0.76 ± 0.04** | 0.03 | 38.4% | 0.03 | 0.96 ± 0.07 |
|  | 20:5 | 529.5 | 16.5 |  | N.D. | N/A | N.D. | N.D. | N.D. | N.D. | N/A | N.D. | N.D. | | N.D. | N.Q. | N/A | N.Q. | N.Q. |
|  | 20:4 | 531.5 | 17.9 |  | 0.00 | 2.4% | 0.00 | 1.24 ± 0.25 | 1.30 ± 0.09 | 0.00 | 1.9% | 0.00 | 0.92 ± 0.09 | | 0.97 ± 0.09 | 0.00 | 2.3% | 0.00 | 1.69 ± 0.67 |
|  | 20:3 | 533.5 | 19.2 |  | N.D. | N/A | N.D. | N.D. | N.D. | 0.00 | 1.6% | 0.00 | 0.74 ± 0.10 | | 1.11 ± 0.09 | 0.00 | 1.3% | 0.00 | 1.12 ± 0.17 |
|  | 22:6 | 555.5 | 16.9 |  | N.Q. | N/A | N.Q. | N.Q. | N.Q. | N.Q. | N/A | N.Q. | N.Q. | | N.Q. | N.Q. | N/A | N.Q. | N.Q. |
|  | 24:2 | 591.5 | 23.7 |  | N.D. | N/A | N.D. | N.D. | N.D. | N.D. | N/A | N.D. | N.D. | | N.D. | N.Q. | N/A | N.Q. | N.Q. |
| **PG** | 16:0/16:1 | 719.6 | 23.5 | **1** | 0.12 | 8.0% | 0.12 | 1.03 ± 0.04 | 1.00 ± 0.02 | 0.02 | 1.6% | 0.02 | 0.98 ± 0.07 | | 0.95 ± 0.04 | 0.04 | 3.7% | 0.04 | 1.15 ± 0.09 |
|  | 18:1/14:0 | 719.6 | 23.4 |  | N.D. | N/A | N.D. | N.D. | N.D. | N.D. | N/A | N.D. | N.D. | | N.D. | N.Q. | N/A | N.Q. | N.Q. |
|  | 16:0/16:0 | 721.5 | 26.5 |  | 0.07 | 4.8% | 0.10 | 1.39 ± 0.04 | 1.32 ± 0.07** | 0.05 | 3.4% | 0.05 | 1.01 ± 0.22 | | 1.04 ± 0.05 | 0.04 | 3.4% | 0.03 | 0.71 ± 0.05 |
|  | 18:1/16:1 | 745.6 | 28.6 |  | 0.06 | 3.9% | 0.05 | 0.92 ± 0.05 | 1.11 ± 0.04** | 0.05 | 3.4% | 0.05 | 0.92 ± 0.05 | | 0.86 ± 0.03** | 0.03 | 2.9% | 0.03 | 1.01 ± 0.06 |
|  | 18:1/16:0 | 747.6 | 30.8 | **2** | 0.24 | 15.8% | 0.21 | 0.88 ± 0.07 | 0.82 ± 0.02** | 0.20 | 12.9% | 0.25 | 1.22 ± 0.20 | | 1.17 ± 0.06 | 0.16 | 14.9% | 0.20 | 1.29 ± 0.11* |
|  | 18:0/16:1 | 747.6 | 28.4 | **3** | 0.52 | 34.5% | 0.47 | 0.90 ± 0.09 | 0.94 ± 0.04 | 0.65 | 41.0% | 0.43 | 0.66 ± 0.14 | | 0.78 ± 0.04** | 0.33 | 31.1% | 0.22 | 0.66 ± 0.06** |
|  | 18:0/16:0 | 749.6 | 30.3 | **4** | 0.04 | 2.9% | 0.10 | 2.22 ± 0.52 | 2.33 ± 0.18** | 0.10 | 6.2% | 0.06 | 0.56 ± 0.08 | | 0.62 ± 0.03** | 0.07 | 6.5% | 0.07 | 0.95 ± 0.03 |
|  | 20:4/16:0 | 769.6 | 25.6 |  | 0.05 | 3.5% | 0.05 | 0.95 ± 0.07 | 1.03 ± 0.04 | 0.04 | 2.5% | 0.04 | 1.10 ± 0.16 | | 0.99 ± 0.05 | 0.02 | 2.3% | 0.03 | 1.19 ± 0.10** |
|  | 18:1/18:1 | 773.5 | 30.4 | **5** | 0.11 | 7.0% | 0.10 | 0.93 ± 0.07 | 1.05 ± 0.06 | 0.10 | 6.0% | 0.11 | 1.16 ± 0.09 | | 1.09 ± 0.05 | 0.11 | 10.0% | 0.14 | 1.36 ± 0.16* |
|  | 18:1/18:0 | 775.6 | 32.3 |  | N.D. | N/A | N.D. | N.D. | N.D. | 0.04 | 2.7% | 0.04 | 0.86 ± 0.14 | | 0.80 ± 0.05** | 0.04 | 3.5% | 0.04 | 1.14 ± 0.07 |
|  | 22:6/16:1 | 791.6 | 25.8 |  | N.D. | N/A | N.D. | N.D. | N.D. | N.D. | N/A | N.D. | N.D. | | N.D. | 0.02 | 1.6% | 0.02 | 0.89 ± 0.04 |
|  | 22:6/16:0 | 793.6 | 26.9 |  | 0.01 | 0.4% | 0.00 | 0.83 ± 0.12 | 0.92 ± 0.04 | 0.01 | 0.6% | 0.01 | 1.12 ± 0.07 | | 0.99 ± 0.06 | 0.01 | 1.1% | 0.01 | 1.29 ± 0.05** |
|  | 20:4/18:1 | 795.6 | 25.9 |  | 0.03 | 2.2% | 0.03 | 0.77 ± 0.05 | 0.67 ± 0.02** | 0.03 | 1.6% | 0.03 | 1.22 ± 0.23 | | 1.17 ± 0.07 | 0.03 | 2.6% | 0.04 | 1.47 ± 0.08** |
|  | 22:5/16:0 | 795.6 | 28.9 |  | N.D. | N/A | N.D. | N.D. | N.D. | N.D. | N/A | N.D. | N.D. | | N.D. | N.Q. | N/A | N.Q. | N.Q. |
|  | 22:4/16:1 | 795.6 | 28.6 |  | 0.02 | 1.2% | 0.03 | 1.74 ± 0.09 | 1.85 ± 0.09** | 0.03 | 1.7% | 0.03 | 0.94 ± 0.20 | | 0.94 ± 0.06 | 0.01 | 1.4% | 0.03 | 1.71 ± 0.20** |
|  | 16:0/22:5 | 795.6 | 28.7 |  | N.Q. | N/A | N.Q. | N.Q. | N.Q. | N.D. | N/A | N.D. | N.D. | | N.D. | 0.00 | 0.1% | 0.00 | 1.06 ± 0.11 |
|  | 20:4/18:0 | 797.6 | 30.6 |  | 0.07 | 4.9% | 0.06 | 0.78 ± 0.06 | 0.86 ± 0.04** | 0.07 | 4.4% | 0.08 | 1.10 ± 0.24 | | 0.96 ± 0.05 | 0.05 | 4.4% | 0.06 | 1.38 ± 0.05** |
|  | 20:4/20:4 | 817.5 | 25.8 |  | 0.02 | 1.0% | 0.01 | 0.76 ± 0.22 | 0.82 ± 0.05** | 0.02 | 0.9% | 0.02 | 1.41 ± 0.26 | | 1.54 ± 0.11** | 0.01 | 0.8% | 0.01 | 1.70 ± 0.14** |
|  | 18:2/22:6 | 817.6 | 25.8 |  | 0.01 | 0.5% | 0.01 | 0.77 ± 0.16 | 0.90 ± 0.05 | 0.01 | 0.6% | 0.01 | 0.90 ± 0.26 | | 1.18 ± 0.07 | 0.00 | 0.4% | 0.01 | 1.28 ± 0.07** |
|  | 18:1/22:6 | 819.6 | 27.4 |  | 0.02 | 1.6% | 0.02 | 0.69 ± 0.14 | 0.71 ± 0.04** | 0.02 | 1.2% | 0.03 | 1.40 ± 0.12 | | 1.12 ± 0.06 | 0.02 | 2.2% | 0.03 | 1.15 ± 0.08 |
|  | 20:4/22:6 | 841.6 | 26.7 |  | 0.03 | 2.1% | 0.01 | 0.43 ± 0.09 | 0.72 ± 0.05 | 0.03 | 2.2% | 0.04 | 1.10 ± 0.21 | | 1.17 ± 0.08 | 0.02 | 2.2% | 0.03 | 1.41 ± 0.09** |
|  | 22:6/22:6 | 865.5 | 26.7 | **6** | 0.07 | 4.8% | 0.07 | 0.99 ± 0.29 | 0.79 ± 0.05** | 0.10 | 6.1% | 0.13 | 1.40 ± 0.24 | | 1.63 ± 0.12** | 0.05 | 4.3% | 0.06 | 1.42 ± 0.03** |
|  | 22:6/22:5 | 867.6 | 26.6 |  | 0.02 | 1.0% | 0.01 | 0.39 ± 0.06 | 0.47 ± 0.03** | 0.01 | 0.9% | 0.02 | 1.10 ± 0.05 | | 1.31 ± 0.14 | 0.01 | 0.5% | 0.01 | 1.03 ± 0.08 |
| **LPI** | 16:1 | 569.6 | 15.3 | **1** | 0.02 | 21.2% | 0.03 | 1.17 ± 0.02 | 1.23 ± 0.03** | 0.02 | 27.8% | 0.02 | 1.04 ± 0.20 | | 0.94 ± 0.03 | 0.02 | 26.3% | 0.03 | 1.53 ± 0.08** |
|  | 16:0 | 571.6 | 18.5 |  | 0.01 | 12.1% | 0.01 | 0.86 ± 0.03 | 0.93 ± 0.04 | 0.01 | 11.3% | 0.01 | 0.99 ± 0.07 | | 1.00 ± 0.04 | 0.01 | 14.7% | 0.01 | 1.14 ± 0.10 |
|  | 18:1 | 597.6 | 18.0 |  | 0.01 | 13.8% | 0.01 | 0.81 ± 0.06 | 0.94 ± 0.03 | 0.01 | 11.4% | 0.01 | 0.82 ± 0.03 | | 0.90 ± 0.04 | 0.01 | 13.9% | 0.01 | 1.00 ± 0.17 |
|  | 18:0 | 599.6 | 20.6 | **2** | 0.03 | 33.2% | 0.03 | 0.81 ± 0.01 | 0.78 ± 0.03** | 0.02 | 24.6% | 0.02 | 0.95 ± 0.05 | | 0.99 ± 0.03 | 0.02 | 18.9% | 0.02 | 1.33 ± 0.07 |
|  | 20:4 | 619.6 | 17.9 | **3** | 0.02 | 19.7% | 0.02 | 0.89 ± 0.05 | 0.99 ± 0.04 | 0.02 | 24.8% | 0.02 | 1.05 ± 0.02 | | 1.07 ± 0.05 | 0.02 | 18.9% | 0.03 | 1.90 ± 0.10** |
|  | 20:3 | 621.6 | 19.2 |  | N.D. | N/A | N.D. | N.D. | N.D. | N.Q. | N/A | N.Q. | N.Q. | | N.Q. | 0.01 | 7.3% | 0.01 | 1.24 ± 0.08 |
|  | 22:6 | 643.6 | 17.9 |  | N.D. | N/A | N.D. | N.D. | N.D. | N.Q. | N/A | N.Q. | N.Q. | | N.Q. | N.Q. | N/A | N.Q. | N.Q. |
| **PI** | 16:0/16:0 | 809.7 | 24.9 |  | 0.09 | 0.6% | 0.14 | 1.56 ± 0.07 | 1.58 ± 0.04** | 0.11 | 0.6% | 0.09 | 0.86 ± 0.04 | | 0.99 ± 0.04 | 0.13 | 0.7% | 0.14 | 1.09 ± 0.02 |
|  | 16:0/20:4 | 857.7 | 24.3 | **1** | 2.50 | 17.8% | 2.89 | 1.17 ± 0.08 | 1.24 ± 0.03 | 1.85 | 10.2% | 1.76 | 0.96 ± 0.08 | | 0.90 ± 0.07 | 3.10 | 17.7% | 4.38 | 1.41 ± 0.04* |
|  | 16:0/20:3 | 859.7 | 25.3 |  | 0.34 | 2.4% | 0.38 | 1.13 ± 0.06 | 1.14 ± 0.03 | 0.26 | 1.4% | 0.23 | 0.90 ± 0.09 | | 0.95 ± 0.06 | 0.42 | 2.4% | 0.49 | 1.16 ± 0.06 |
|  | 18:1/18:1 | 861.5 | 26.7 |  | 0.10 | 0.7% | 0.14 | 1.47 ± 0.07 | 1.26 ± 0.03** | 0.11 | 0.6% | 0.07 | 0.69 ± 0.06 | | 0.65 ± 0.03** | 0.17 | 1.0% | 0.16 | 0.93 ± 0.03 |
|  | 18:0/18:2 | 861.7 | 27.3 |  | 0.05 | 0.3% | 0.06 | 1.33 ± 0.20 | 1.61 ± 0.13** | 0.06 | 0.3% | 0.04 | 0.69 ± 0.04 | | 0.79 ± 0.05 | 0.06 | 0.3% | 0.10 | 1.72 ± 0.06* |
|  | 18:0/18:1 | 863.7 | 29.8 |  | 0.37 | 2.7% | 0.49 | 1.31 ± 0.07 | 1.26 ± 0.02** | 0.38 | 2.1% | 0.24 | 0.62 ± 0.02 | | 0.67 ± 0.03** | 0.99 | 5.6% | 0.78 | 0.79 ± 0.02* |
|  | 16:0/22:6 | 881.7 | 25.2 |  | 0.39 | 2.8% | 0.44 | 1.12 ± 0.07 | 1.26 ± 0.04** | 0.26 | 1.4% | 0.20 | 0.76 ± 0.12 | | 0.79 ± 0.04 | 0.47 | 2.7% | 0.73 | 1.54 ± 0.12** |
|  | 18:1/20:4 | 883.7 | 25.7 | **2** | 2.59 | 18.5% | 4.00 | 1.54 ± 0.11 | 1.38 ± 0.05** | 2.27 | 12.5% | 2.47 | 1.09 ± 0.10 | | 1.04 ± 0.08 | 2.69 | 15.4% | 4.57 | 1.70 ± 0.05** |
|  | 18:0/20:4 | 885.7 | 27.0 | **3** | 6.14 | 43.7% | 13.42 | 2.19 ± 0.24 | 1.75 ± 0.05 | 11.22 | 61.9% | 5.99 | 0.53 ± 0.07 | | 0.54 ± 0.04** | 7.43 | 42.4% | 11.26 | 1.51 ± 0.07 |
|  | 18:0/20:3 | 887.7 | 27.5 | **4** | 0.97 | 6.9% | 2.13 | 2.20 ± 0.08 | 2.08 ± 0.09** | 1.14 | 6.3% | 0.76 | 0.67 ± 0.04 | | 0.61 ± 0.03** | 1.21 | 6.9% | 1.70 | 1.40 ± 0.04** |
|  | 18:0/20:1 | 891.7 | 29.7 |  | N.D. | N/A | N.D. | N.D. | N.D. | N.Q. | N/A | N.Q. | N.Q. | | N.Q. | N.D. | N/A | N.D. | N.D. |
|  | 20:4/20:4 | 905.7 | 24.3 |  | 0.03 | 0.2% | 0.03 | 1.12 ± 0.17 | 1.70 ± 0.05** | 0.08 | 0.4% | 0.03 | 0.45 ± 0.04 | | 0.50 ± 0.01** | 0.05 | 0.3% | 0.10 | 2.00 ± 0.13 |
|  | 18:1/22:6 | 907.7 | 25.3 |  | 0.06 | 0.4% | 0.09 | 1.56 ± 0.29 | 1.76 ± 0.09** | 0.04 | 0.2% | 0.04 | 0.94 ± 0.02 | | 0.81 ± 0.05** | 0.08 | 0.5% | 0.10 | 1.22 ± 0.06* |
|  | 20:4/20:3 | 907.7 | 24.5 |  | 0.00 | 0.0% | 0.01 | 3.32 ± 0.07 | 3.13 ± 0.26 | 0.02 | 0.1% | 0.02 | 0.97 ± 0.18 | | 0.91 ± 0.06 | 0.03 | 0.1% | 0.05 | 1.90 ± 0.13** |
|  | 18:0/22:6 | 909.7 | 26.9 |  | 0.24 | 1.7% | 0.49 | 2.07 ± 0.08 | 2.02 ± 0.06** | 0.18 | 1.0% | 0.18 | 0.96 ± 0.08 | | 0.95 ± 0.06 | 0.34 | 1.9% | 0.45 | 1.34 ± 0.02** |
|  | 20:2/20:4 | 909.7 | 26.0 |  | 0.02 | 0.2% | 0.03 | 1.19 ± 0.07 | 1.30 ± 0.06** | 0.02 | 0.1% | 0.03 | 1.27 ± 0.25 | | 1.07 ± 0.04 | 0.03 | 0.1% | 0.03 | 1.17 ± 0.11 |
|  | 20:1/20:4 | 911.7 | 27.5 |  | 0.02 | 0.1% | 0.05 | 2.62 ± 0.12 | 2.67 ± 0.11** | 0.02 | 0.1% | 0.02 | 0.80 ± 0.09 | | 0.77 ± 0.03** | 0.04 | 0.2% | 0.04 | 1.08 ± 0.08 |
|  | 18:0/22:5 | 911.7 | 27.4 |  | 0.06 | 0.4% | 0.09 | 1.71 ± 0.14 | 1.88 ± 0.06** | 0.04 | 0.2% | 0.02 | 0.70 ± 0.02 | | 0.70 ± 0.02** | 0.08 | 0.5% | 0.11 | 1.28 ± 0.02 |
|  | 18:0/22:4 | 913.7 | 28.7 |  | 0.09 | 0.7% | 0.11 | 1.21 ± 0.24 | 1.11 ± 0.05 | 0.08 | 0.4% | 0.05 | 0.65 ± 0.06 | | 0.68 ± 0.02** | 0.19 | 1.1% | 0.21 | 1.11 ± 0.11 |
|  | 22:6/20:4 | 929.7 | 24.1 |  | N.Q. | N/A | N.Q. | N.Q. | N.Q. | N.Q. | N/A | N.Q. | N.Q. | | N.Q. | N.Q. | N/A | N.Q. | N.Q. |
| **LPS** | 16:0 | 496.5 | 20.0 | **1** | 0.08 | 9.3% | 0.07 | 0.90 ± 0.06 | 1.00 ± 0.03 | 0.07 | 8.9% | 0.07 | 0.99 ± 0.12 | | 1.06 ± 0.04 | 0.08 | 14.1% | 0.09 | 1.12 ± 0.15 |
|  | 18:1 | 522.5 | 20.4 | **2** | 0.08 | 9.0% | 0.08 | 1.04 ± 0.08 | 1.16 ± 0.05 | 0.08 | 9.8% | 0.07 | 0.89 ± 0.06 | | 0.91 ± 0.03** | 0.07 | 11.5% | 0.09 | 1.29 ± 0.07* |
|  | 18:0 | 524.5 | 22.0 | **3** | 0.34 | 40.2% | 0.33 | 0.97 ± 0.07 | 1.02 ± 0.04 | 0.32 | 40.6% | 0.29 | 0.90 ± 0.05 | | 1.05 ± 0.03 | 0.16 | 26.4% | 0.28 | 1.78 ± 0.14* |
|  | 20:6 | 540.5 | 17.2 |  | 0.03 | 3.4% | 0.03 | 0.91 ± 0.12 | 0.90 ± 0.06* | 0.02 | 2.9% | 0.02 | 0.93 ± 0.14 | | 0.89 ± 0.06 | 0.02 | 3.8% | 0.02 | 0.98 ± 0.11 |
|  | 20:4 | 544.5 | 19.8 |  | 0.02 | 2.6% | 0.02 | 1.04 ± 0.07 | 1.16 ± 0.04** | 0.02 | 2.9% | 0.02 | 1.00 ± 0.16 | | 1.05 ± 0.04 | 0.02 | 3.5% | 0.03 | 1.54 ± 0.09* |
|  | 20:3 | 546.5 | 21.9 |  | 0.03 | 3.5% | 0.03 | 0.94 ± 0.11 | 0.89 ± 0.05 | 0.02 | 3.0% | 0.03 | 1.08 ± 0.06 | | 1.11 ± 0.04 | 0.02 | 4.1% | 0.02 | 0.96 ± 0.07 |
|  | 20:1 | 550.5 | 22.1 |  | N.Q. | N/A | N.Q. | N.Q. | N.Q. | 0.01 | 1.1% | 0.01 | 0.78 ± 0.10 | | 0.74 ± 0.05** | N.D. | N/A | N.D. | N.D. |
|  | 22:6 | 568.5 | 19.6 | **4** | 0.20 | 23.3% | 0.20 | 0.99 ± 0.15 | 1.09 ± 0.06* | 0.17 | 21.7% | 0.17 | 1.03 ± 0.05 | | 1.07 ± 0.05 | 0.17 | 28.0% | 0.26 | 1.57 ± 0.08** |
|  | 22:5 | 570.5 | 19.8 |  | 0.02 | 2.2% | 0.02 | 0.96 ± 0.20 | 1.04 ± 0.06 | 0.02 | 2.5% | 0.02 | 1.21 ± 0.08 | | 1.24 ± 0.07* | 0.02 | 2.7% | 0.02 | 1.19 ± 0.10 |
|  | 22:4 | 572.5 | 21.3 |  | 0.02 | 1.8% | 0.02 | 1.32 ± 0.14 | 1.29 ± 0.07** | 0.02 | 2.4% | 0.02 | 1.09 ± 0.17 | | 1.18 ± 0.07** | 0.01 | 2.4% | 0.03 | 2.12 ± 0.30 |
|  | 24:0 | 608.5 | 23.8 |  | 0.04 | 4.8% | 0.04 | 0.98 ± 0.09 | 0.87 ± 0.04 | 0.03 | 4.0% | 0.03 | 0.96 ± 0.17 | | 0.98 ± 0.05 | 0.02 | 3.5% | 0.04 | 2.12 ± 0.22 |
| **PS** | 16:1/18:1 | 758.5 | 24.2 |  | 0.15 | 0.1% | 0.14 | 0.96 ± 0.11 | 0.94 ± 0.04 | 0.15 | 0.2% | 0.22 | 1.49 ± 0.13 | | 1.09 ± 0.05 | 0.14 | 0.2% | 0.23 | 1.60 ± 0.14** |
|  | 16:0/18:1 | 760.5 | 25.5 |  | 1.62 | 1.4% | 1.43 | 0.88 ± 0.05 | 0.91 ± 0.03** | 1.52 | 1.9% | 1.52 | 1.00 ± 0.09 | | 1.01 ± 0.05 | 1.80 | 2.3% | 1.71 | 0.95 ± 0.06 |
|  | 18:0/16:1 | 760.5 | 25.5 |  | 0.15 | 0.1% | 0.16 | 1.02 ± 0.05 | 0.99 ± 0.04 | 0.15 | 0.2% | 0.16 | 1.11 ± 0.14 | | 1.08 ± 0.08 | 0.09 | 0.1% | 0.18 | 1.94 ± 0.26** |
|  | 18:0/16:0 | 762.5 | 27.1 | **1** | 4.84 | 4.1% | 3.16 | 0.65 ± 0.11 | 0.70 ± 0.03** | 5.59 | 7.0% | 5.04 | 0.90 ± 0.08 | | 0.95 ± 0.06 | 5.75 | 7.4% | 8.57 | 1.49 ± 0.02** |
|  | 16:0/20:4 | 782.5 | 24.4 |  | 0.43 | 0.4% | 0.34 | 0.79 ± 0.08 | 0.80 ± 0.02** | 0.58 | 0.7% | 0.70 | 1.21 ± 0.12 | | 1.14 ± 0.07 | 0.23 | 0.3% | 0.28 | 1.24 ± 0.12 |
|  | 18:1/18:2 | 784.5 | 25.1 |  | 0.71 | 0.6% | 0.93 | 1.32 ± 0.06 | 1.49 ± 0.09** | 0.82 | 1.0% | 0.83 | 1.01 ± 0.08 | | 1.03 ± 0.05 | 1.92 | 2.5% | 2.41 | 1.26 ± 0.11 |
|  | 16:0/20:3 | 784.5 | 25.1 |  | N.Q. | N/A | N.Q. | N.Q. | N.Q. | N.Q. | N/A | N.Q. | N.Q. | | N.Q. | N.Q. | N/A | N.Q. | N.Q. |
|  | 18:1/18:1 | 786.5 | 25.6 | **2** | 7.08 | 6.0% | 5.80 | 0.82 ± 0.05 | 0.96 ± 0.05 | 5.24 | 6.6% | 5.72 | 1.09 ± 0.13 | | 1.00 ± 0.04 | 9.78 | 12.5% | 11.67 | 1.19 ± 0.07* |
|  | 18:0/18:1 | 788.5 | 27.6 | **3** | 9.80 | 8.3% | 9.93 | 1.01 ± 0.10 | 1.02 ± 0.03 | 6.08 | 7.6% | 5.90 | 0.97 ± 0.06 | | 1.02 ± 0.04 | 6.12 | 7.8% | 7.21 | 1.18 ± 0.04** |
|  | 16:0/22:6 | 806.5 | 24.2 |  | 0.18 | 0.2% | 0.14 | 0.76 ± 0.08 | 0.83 ± 0.03** | 1.15 | 1.4% | 1.61 | 1.40 ± 0.13 | | 1.28 ± 0.08** | 0.09 | 0.1% | 0.11 | 1.28 ± 0.09 |
|  | 18:1/20:4 | 808.5 | 24.7 |  | 1.12 | 0.9% | 1.03 | 0.92 ± 0.06 | 0.94 ± 0.03* | 1.16 | 1.5% | 1.51 | 1.30 ± 0.17 | | 1.19 ± 0.09** | 0.79 | 1.0% | 0.79 | 1.00 ± 0.09 |
|  | 16:0/22:5 | 808.5 | 24.7 |  | N.Q. | N/A | N.Q. | N.Q. | N.Q. | 0.17 | 0.2% | 0.28 | 1.71 ± 0.31 | | 1.42 ± 0.06** | N.D. | N/A | N.D. | N.D. |
|  | 18:0/20:4 | 810.5 | 26.1 | **4** | 4.00 | 3.4% | 4.05 | 1.01 ± 0.05 | 1.07 ± 0.04 | 4.32 | 5.4% | 3.79 | 0.88 ± 0.18 | | 0.87 ± 0.04** | 1.08 | 1.4% | 1.15 | 1.06 ± 0.06 |
|  | 18:0/20:3 | 812.5 | 26.9 |  | 1.10 | 0.9% | 0.93 | 0.84 ± 0.04 | 1.12 ± 0.06* | 1.04 | 1.3% | 1.07 | 1.03 ± 0.14 | | 0.98 ± 0.05 | 0.96 | 1.2% | 0.98 | 1.03 ± 0.04* |
|  | 18:1/20:2 | 812.5 | 26.6 |  | 0.06 | 0.0% | 0.05 | 0.85 ± 0.23 | 1.25 ± 0.11** | 0.05 | 0.1% | 0.06 | 1.15 ± 0.16 | | 0.14 ± 0.04** | 0.19 | 0.2% | 0.20 | 1.08 ± 0.10 |
|  | 20:1/18:1 | 814.5 | 28.6 |  | 0.42 | 0.4% | 0.38 | 0.90 ± 0.08 | 1.07 ± 0.04* | 0.25 | 0.3% | 0.25 | 1.00 ± 0.07 | | 1.12 ± 0.07 | 0.34 | 0.4% | 0.42 | 1.26 ± 0.11 |
|  | 18:0/20:2 | 814.5 | 28.6 |  | 0.10 | 0.1% | 0.10 | 0.98 ± 0.04 | 0.99 ± 0.06 | 0.08 | 0.1% | 0.07 | 0.85 ± 0.14 | | 0.96 ± 0.08 | 0.06 | 0.1% | 0.12 | 2.05 ± 0.21 |
|  | 18:0/20:1 | 816.5 | 30.0 |  | N.Q. | N/A | N.Q. | N.Q. | N.Q. | N.D. | N/A | N.D. | N.D. | | N.D. | N.Q. | N/A | N.Q. | N.Q. |
|  | 20:0/18:1 | 816.5 | 30.0 |  | 0.37 | 0.3% | 0.31 | 0.83 ± 0.04 | 0.82 ± 0.03** | 0.34 | 0.4% | 0.21 | 0.62 ± 0.16 | | 0.74 ± 0.05** | 0.54 | 0.7% | 0.48 | 0.90 ± 0.08 |
|  | 18:1/22:6 | 832.5 | 24.5 | **5** | 3.85 | 3.2% | 3.16 | 0.82 ± 0.06 | 0.83 ± 0.03** | 3.48 | 4.4% | 3.57 | 1.02 ± 0.11 | | 1.11 ± 0.08 | 0.52 | 0.7% | 0.61 | 1.17 ± 0.09 |
|  | 18:0/22:6 | 834.5 | 25.7 | **6** | 64.05 | 54.0% | 63.63 | 0.99 ± 0.07 | 1.00 ± 0.02 | 34.93 | 43.7% | 52.98 | 1.52 ± 0.16 | | 1.35 ± 0.06** | 43.29 | 55.4% | 53.62 | 1.24 ± 0.06** |
|  | 18:1/22:5 | 834.5 | 25.7 |  | 1.03 | 0.9% | 0.73 | 0.72 ± 0.12 | 0.79 ± 0.05 | 0.37 | 0.5% | 0.58 | 1.57 ± 0.36 | | 1.40 ± 0.09** | 0.10 | 0.1% | 0.10 | 0.95 ± 0.09 |
|  | 18:0/22:5 | 836.5 | 27.2 | **7** | 8.63 | 7.3% | 8.31 | 0.96 ± 0.06 | 0.98 ± 0.04 | 5.34 | 6.7% | 6.76 | 1.27 ± 0.18 | | 1.28 ± 0.08** | 0.32 | 0.4% | 0.43 | 1.35 ± 0.04** |
|  | 18:0/22:4 | 838.5 | 27.6 | **8** | 3.79 | 3.2% | 3.46 | 0.91 ± 0.09 | 0.90 ± 0.03* | 3.09 | 3.9% | 3.31 | 1.07 ± 0.11 | | 0.95 ± 0.04 | 1.60 | 2.0% | 2.20 | 1.38 ± 0.04** |
|  | 22:1/18:1 | 842.5 | 30.9 |  | N.Q. | N/A | N.Q. | N.Q. | N.Q. | N.Q. | N/A | N.Q. | N.Q. | | N.Q. | N.D. | N/A | N.D. | N.D. |
|  | 22:0/18:1 | 844.5 | 33.3 |  | N.Q. | N/A | N.Q. | N.Q. | N.Q. | N.Q. | N/A | N.Q. | N.Q. | | N.Q. | N.Q. | N/A | N.Q. | N.Q. |
|  | 18:0/22:1 | 844.5 | 33.3 |  | N.Q. | N/A | N.Q. | N.Q. | N.Q. | N.D. | N/A | N.D. | N.D. | | N.D. | 0.18 | 0.2% | 0.18 | 0.95 ± 0.10 |
|  | 20:4/22:6 | 854.5 | 23.2 |  | 0.64 | 0.5% | 0.33 | 0.51 ± 0.14 | 0.50 ± 0.04** | 0.39 | 0.5% | 0.63 | 1.61 ± 0.27 | | 1.37 ± 0.07** | 0.05 | 0.1% | 0.09 | 1.69 ± 0.08* |
|  | 22:6/20:0 | 862.5 | 27.1 |  | N.D. | N/A | N.D. | N.D. | N.D. | N.D. | N/A | N.D. | N.D. | | N.D. | 0.05 | 0.1% | 0.13 | 2.53 ± 0.45 |
|  | 18:0/24:6 | 862.5 | 27.3 |  | N.Q. | N/A | N.Q. | N.Q. | N.Q. | 0.18 | 0.2% | 0.23 | 1.28 ± 0.13 | | 1.06 ± 0.06 | 0.19 | 0.2% | 0.34 | 1.82 ± 0.09 |
|  | 18:0/24:4 | 866.5 | 26.2 |  | N.D. | N/A | N.D. | N.D. | N.D. | N.Q. | N/A | N.Q. | N.Q. | | N.Q. | 0.22 | 0.3% | 0.25 | 1.14 ± 0.15 |
|  | 24:1/18:1 | 870.5 | 33.2 |  | N.Q. | N/A | N.Q. | N.Q. | N.Q. | N.Q. | N/A | N.Q. | N.Q. | | N.Q. | N.Q. | N/A | N.Q. | N.Q. |
|  | 24:0/18:1 | 872.5 | 36.8 |  | N.Q. | N/A | N.Q. | N.Q. | N.Q. | N.Q. | N/A | N.Q. | N.Q. | | N.Q. | N.Q. | N/A | N.Q. | N.Q. |
|  | 22:6/22:6 | 878.5 | 23.1 |  | 1.52 | 1.3% | 1.00 | 0.66 ± 0.09 | 0.59 ± 0.04** | 1.09 | 1.4% | 1.28 | 1.17 ± 0.13 | | 1.24 ± 0.08** | 1.07 | 1.4% | 1.35 | 1.26 ± 0.09 |
|  | 22:5/22:6 | 880.5 | 23.6 |  | 0.31 | 0.3% | 0.14 | 0.45 ± 0.12 | 0.65 ± 0.05** | 0.21 | 0.3% | 0.25 | 1.20 ± 0.23 | | 1.22 ± 0.08* | 0.20 | 0.2% | 0.32 | 1.59 ± 0.12** |
|  | 22:4/22:6 | 882.5 | 24.6 |  | 2.67 | 2.3% | 2.90 | 1.09 ± 0.28 | 1.23 ± 0.08 | 2.16 | 2.7% | 3.45 | 1.60 ± 0.19 | | 1.17 ± 0.10** | 0.52 | 0.7% | 0.60 | 1.16 ± 0.08* |
| **LPA** | 14:0 | 381.5 | 14.7 |  | 0.13 | 2.0% | 0.12 | 0.94 ± 0.18 | 0.66 ± 0.06** | 0.06 | 1.4% | 0.03 | 0.59 ± 0.07 | | 0.78 ± 0.06 | 0.20 | 4.9% | 0.22 | 1.11 ± 0.12 |
|  | 16:0 | 409.5 | 15.4 | **1** | 0.74 | 11.4% | 0.46 | 0.62 ± 0.03 | 0.62 ± 0.02** | 0.50 | 12.0% | 0.51 | 1.01 ± 0.10 | | 1.06 ± 0.03** | 0.38 | 9.3% | 0.46 | 1.22 ± 0.24 |
|  | 18:2 | 433.5 | 16.8 |  | 0.05 | 0.8% | 0.05 | 0.97 ± 0.17 | 1.04 ± 0.05 | 0.03 | 0.6% | 0.04 | 1.58 ± 0.46 | | 1.44 ± 0.12** | 0.05 | 1.2% | 0.05 | 1.11 ± 0.16 |
|  | 18:1 | 435.5 | 17.8 | **2** | 1.47 | 22.7% | 1.03 | 0.70 ± 0.07 | 0.71 ± 0.06** | 1.07 | 25.5% | 1.29 | 1.20 ± 0.13 | | 1.15 ± 0.05** | 1.05 | 26.0% | 1.00 | 0.95 ± 0.08 |
|  | 18:0 | 437.5 | 19.3 | **3** | 2.72 | 41.9% | 1.35 | 0.50 ± 0.11 | 0.48 ± 0.05** | 1.37 | 32.5% | 1.42 | 1.04 ± 0.11 | | 1.10 ± 0.05 | 1.43 | 35.3% | 1.40 | 0.98 ± 0.08 |
|  | 20:6 | 453.5 | 13.9 |  | 0.37 | 5.8% | 0.27 | 0.72 ± 0.12 | 0.77 ± 0.05** | 0.19 | 4.5% | 0.16 | 0.82 ± 0.11 | | 0.84 ± 0.05** | 0.32 | 7.8% | 0.33 | 1.03 ± 0.11 |
|  | 20:4 | 457.5 | 15.2 |  | 0.25 | 3.9% | 0.30 | 1.21 ± 0.08 | 1.18 ± 0.05** | 0.25 | 6.0% | 0.32 | 1.26 ± 0.17 | | 1.17 ± 0.04** | 0.25 | 6.1% | 0.32 | 1.31 ± 0.12* |
|  | 20:1 | 463.5 | 19.3 |  | 0.18 | 2.7% | 0.20 | 1.14 ± 0.53 | 1.26 ± 0.09** | 0.21 | 5.0% | 0.26 | 1.24 ± 0.11 | | 1.14 ± 0.06 | 0.12 | 2.9% | 0.12 | 0.99 ± 0.06 |
|  | 22:6 | 481.5 | 15.3 |  | 0.41 | 6.4% | 0.40 | 0.96 ± 0.05 | 1.05 ± 0.05 | 0.40 | 9.4% | 0.47 | 1.19 ± 0.12 | | 1.20 ± 0.04** | 0.15 | 3.6% | 0.58 | 3.98 ± 0.31* |
|  | 22:4 | 485.5 | 16.3 |  | 0.16 | 2.4% | 0.13 | 0.84 ± 0.06 | 0.84 ± 0.05 | 0.13 | 3.1% | 0.15 | 1.11 ± 0.25 | | 1.22 ± 0.06** | 0.12 | 2.9% | 0.21 | 1.79 ± 0.16* |
| **PA** | 16:0/18:1 | 673.5 | 25.0 |  | N.Q. | N/A | N.Q. | N.Q. | N.Q. | N.Q. | N/A | N.Q. | N.Q. | | N.Q. | N.Q. | N/A | N.Q. | N.Q. |
|  | 18:0/16:1 | 673.5 | 25.0 |  | 0.00 | 0.3% | 0.00 | 1.44 ± 0.40 | 1.16 ± 0.13 | N.Q. | N/A | N.Q. | N.Q. | | N.Q. | 0.00 | 0.4% | 0.00 | 1.62 ± 0.12** |
|  | 16:0/20:4 | 695.5 | 24.0 |  | N.Q. | N/A | N.Q. | N.Q. | N.Q. | 0.00 | 0.9% | 0.00 | 0.76 ± 0.15 | | 0.93 ± 0.09 | N.D. | N/A | N.D. | N.D. |
|  | 16:0/20:2 | 699.5 | 26.4 |  | 0.00 | 0.5% | 0.00 | 0.85 ± 0.23 | 1.02 ± 0.11 | N.D. | N/A | N.D. | N.D. | | N.D. | 0.00 | 0.2% | 0.00 | 1.74 ± 0.32 |
|  | 18:1/18:1 | 699.5 | 26.3 |  | N.Q. | N/A | N.Q. | N.Q. | N.Q. | 0.02 | 5.2% | 0.02 | 0.83 ± 0.19 | | 0.86 ± 0.05 | 0.02 | 4.0% | 0.02 | 1.04 ± 0.08 |
|  | 18:0/18:2 | 699.5 | 26.1 |  | 0.00 | 0.4% | 0.00 | 1.09 ± 0.16 | 1.00 ± 0.07 | N.Q. | N/A | N.Q. | N.Q. | | N.Q. | 0.00 | 0.1% | 0.00 | 1.41 ± 0.18* |
|  | 18:0/18:1 | 701.5 | 28.9 | **1** | 0.10 | 14.4% | 0.11 | 1.07 ± 0.09 | 1.02 ± 0.04 | 0.03 | 7.7% | 0.02 | 0.69 ± 0.15 | | 0.64 ± 0.06** | 0.12 | 22.5% | 0.13 | 1.03 ± 0.08 |
|  | 16:0/20:1 | 701.5 | 28.4 |  | 0.04 | 5.2% | 0.04 | 1.04 ± 0.18 | 0.88 ± 0.06** | N.D. | N/A | N.D. | N.D. | | N.D. | 0.01 | 2.7% | 0.01 | 0.96 ± 0.10 |
|  | 16:0/22:6 | 719.5 | 23.3 | **2** | 0.08 | 11.3% | 0.10 | 1.26 ± 0.16 | 1.24 ± 0.05** | N.Q. | N/A | N.Q. | N.Q. | | N.Q. | 0.08 | 14.1% | 0.12 | 1.63 ± 0.17* |
|  | 16:0/22:5 | 721.5 | 24.4 |  | N.D. | N/A | N.D. | N.D. | N.D. | 0.02 | 4.2% | 0.02 | 1.00 ± 0.23 | | 1.10 ± 0.08 | N.Q. | N/A | N.Q. | N.Q. |
|  | 18:1/20:4 | 721.5 | 24.4 |  | N.D. | N/A | N.D. | N.D. | N.D. | 0.01 | 1.2% | 0.00 | 0.89 ± 0.10 | | 0.87 ± 0.06** | 0.00 | 0.8% | 0.00 | 1.09 ± 0.10 |
|  | 16:0/22:4 | 723.5 | 26.7 |  | 0.01 | 1.6% | 0.01 | 0.46 ± 0.07 | 0.47 ± 0.03** | N.Q. | N/A | N.Q. | N.Q. | | N.Q. | 0.00 | 0.7% | 0.00 | 1.22 ± 0.12 |
|  | 18:0/20:4 | 723.5 | 26.7 | **3** | 0.10 | 14.7% | 0.10 | 1.01 ± 0.30 | 1.01 ± 0.04 | 0.03 | 5.8% | 0.01 | 0.50 ± 0.12 | | 0.62 ± 0.05** | 0.04 | 7.5% | 0.05 | 1.15 ± 0.04* |
|  | 18:1/22:6 | 745.5 | 24.7 | **4** | 0.16 | 23.4% | 0.20 | 1.20 ± 0.02 | 1.17 ± 0.04** | 0.02 | 4.9% | 0.02 | 1.03 ± 0.08 | | 0.93 ± 0.04* | 0.17 | 32.5% | 0.21 | 1.23 ± 0.06 |
|  | 18:0/22:6 | 747.5 | 24.6 | **5** | 0.17 | 24.0% | 0.27 | 1.61 ± 0.12 | 1.61 ± 0.08** | 0.29 | 64.0% | 0.15 | 0.52 ± 0.04 | | 0.68 ± 0.05** | 0.07 | 12.1% | 0.09 | 1.31 ± 0.08** |
|  | 16:0/24:6 | 747.5 | 24.6 |  | 0.03 | 4.1% | 0.02 | 0.75 ± 0.04 | 0.88 ± 0.04** | 0.03 | 6.1% | 0.03 | 1.08 ± 0.10 | | 1.22 ± 0.05** | 0.01 | 2.4% | 0.01 | 1.15 ± 0.06 |
| **SM** | d18:1/16:1 | 701.5 | 28.8 |  | 0.21 | 0.2% | 0.16 | 0.76 ± 0.06 | 0.81 ± 0.03 | 0.15 | 0.4% | 0.15 | 0.98 ± 0.18 | | 0.99 ± 0.07 | 0.23 | 0.2% | 0.34 | 1.49 ± 0.17 |
|  | d18:1/16:0 | 703.5 | 28.9 |  | 1.05 | 1.1% | 1.11 | 1.05 ± 0.11 | 0.96 ± 0.03 | 0.93 | 2.5% | 1.07 | 1.16 ± 0.16 | | 1.11 ± 0.08 | 1.56 | 1.4% | 2.01 | 1.29 ± 0.16 |
|  | d18:1/18:1 | 729.5 | 30.3 | **1** | 5.50 | 5.7% | 3.72 | 0.68 ± 0.13 | 0.67 ± 0.02** | 3.52 | 9.4% | 3.16 | 0.90 ± 0.14 | | 0.85 ± 0.04** | 5.04 | 4.7% | 8.02 | 1.59 ± 0.26 |
|  | d18:1/18:0 | 731.5 | 32.8 | **2** | 17.95 | 18.6% | 11.27 | 0.63 ± 0.10 | 0.58 ± 0.02** | 8.14 | 21.8% | 10.18 | 1.25 ± 0.11 | | 1.14 ± 0.08** | 13.81 | 12.8% | 21.14 | 1.53 ± 0.17** |
|  | d18:1/20:6 | 747.5 | 27.9 |  | 3.54 | 3.7% | 2.79 | 0.79 ± 0.06 | 0.74 ± 0.03** | N.Q. | N/A | N.Q. | N.Q. | | N.Q. | 3.33 | 3.1% | 5.99 | 1.80 ± 0.23* |
|  | d18:1/20:2 | 755.5 | 32.6 |  | 0.11 | 0.1% | 0.08 | 0.74 ± 0.06 | 0.84 ± 0.02** | 0.04 | 0.1% | 0.06 | 1.41 ± 0.14 | | 1.44 ± 0.05** | 0.11 | 0.1% | 0.13 | 1.15 ± 0.11 |
|  | d18:1/20:0 | 759.5 | 33.1 | **3** | 9.85 | 10.2% | 5.88 | 0.60 ± 0.11 | 0.67 ± 0.03** | 2.97 | 7.9% | 2.42 | 0.81 ± 0.24 | | 0.85 ± 0.06 | 10.47 | 9.7% | 19.05 | 1.82 ± 0.21* |
|  | d18:1/22:6 | 775.5 | 27.4 |  | 2.53 | 2.6% | 2.01 | 0.79 ± 0.05 | 0.78 ± 0.04** | 1.02 | 2.7% | 1.14 | 1.11 ± 0.07 | | 0.96 ± 0.05 | 3.18 | 3.0% | 2.71 | 0.85 ± 0.09 |
|  | d18:1/22:3 | 781.5 | 34.0 |  | 1.02 | 1.1% | 0.71 | 0.70 ± 0.04 | 0.83 ± 0.05** | 0.73 | 2.0% | 0.66 | 0.90 ± 0.10 | | 0.98 ± 0.05 | 0.69 | 0.6% | 1.20 | 1.74 ± 0.18** |
|  | d18:1/22:2 | 783.5 | 34.6 | **4** | 15.87 | 16.4% | 7.11 | 0.45 ± 0.08 | 0.42 ± 0.01** | 7.11 | 19.0% | 6.72 | 0.94 ± 0.13 | | 0.87 ± 0.03** | 21.13 | 19.6% | 24.20 | 1.14 ± 0.08 |
|  | d18:1/22:0 | 787.5 | 35.6 | **5** | 6.78 | 7.0% | 4.83 | 0.71 ± 0.02 | 0.71 ± 0.03** | 0.71 | 1.9% | 1.03 | 1.45 ± 0.15 | | 1.43 ± 0.07** | 8.80 | 8.2% | 13.96 | 1.58 ± 0.17 |
|  | d18:1/24:4 | 807.5 | 33.2 | **6** | 14.71 | 15.2% | 10.58 | 0.72 ± 0.18 | 0.69 ± 0.03** | 7.06 | 18.9% | 10.40 | 1.47 ± 0.07 | | 1.36 ± 0.10** | 14.44 | 13.4% | 17.02 | 1.18 ± 0.11 |
|  | d18:1/24:1 | 813.5 | 35.7 | **7** | 11.66 | 12.1% | 4.15 | 0.36 ± 0.01 | 0.38 ± 0.01** | 3.25 | 8.7% | 4.14 | 1.27 ± 0.09 | | 1.04 ± 0.05 | 14.19 | 13.2% | 29.65 | 2.09 ± 0.15** |
|  | d18:1/24:0 | 815.5 | 39.6 | **8** | 5.83 | 6.0% | 2.14 | 0.37 ± 0.02 | 0.36 ± 0.02** | 1.78 | 4.8% | 1.98 | 1.11 ± 0.16 | | 1.00 ± 0.07 | 10.79 | 10.0% | 20.89 | 1.94 ± 0.20 |
| **Cer** | d18:1/16:1 | 536.5 | 31.4 | **1** | 2.02 | 5.6% | 1.94 | 0.96 ± 0.02 | 1.06 ± 0.04 | 10.92 | 38.1% | 10.10 | 0.92 ± 0.17 | | 0.94 ± 0.06 | 1.61 | 2.6% | 0.96 | 0.60 ± 0.05 |
|  | d18:1/18:1 | 564.5 | 34.1 |  | N.Q. | N/A | N.Q. | N.Q. | N.Q. | 0.20 | 0.7% | 0.30 | 1.47 ± 0.10 | | 1.42 ± 0.05** | 0.44 | 0.7% | 0.49 | 1.10 ± 0.13 |
|  | d18:1/18:0 | 566.5 | 36.8 | **2** | 5.80 | 16.0% | 4.97 | 0.86 ± 0.09 | 0.94 ± 0.03 | 4.68 | 16.3% | 5.38 | 1.15 ± 0.18 | | 0.89 ± 0.06 | 5.79 | 9.4% | 3.06 | 0.53 ± 0.06* |
|  | d18:0/18:1 | 566.5 | 36.8 | **3** | 6.13 | 16.9% | 5.55 | 0.90 ± 0.06 | 1.05 ± 0.03 | N.D. | N/A | N.D. | N.D. | | N.D. | 5.70 | 9.3% | 3.00 | 0.53 ± 0.05** |
|  | d18:1/20:4 | 586.5 | 32.5 |  | 0.45 | 1.2% | 0.24 | 0.54 ± 0.04 | 0.59 ± 0.03** | 0.20 | 0.7% | 0.35 | 1.71 ± 0.27 | | 1.63 ± 0.10** | 0.61 | 1.0% | 0.79 | 1.28 ± 0.11* |
|  | d18:1/20:1 | 592.5 | 37.2 |  | N.Q. | N/A | N.Q. | N.Q. | N.Q. | 0.53 | 1.8% | 0.50 | 0.95 ± 0.04 | | 1.05 ± 0.04 | N.Q. | N/A | N.Q. | N.Q. |
|  | d18:1/20:0 | 594.5 | 37.6 |  | 0.53 | 1.5% | 0.34 | 0.65 ± 0.03 | 0.85 ± 0.04 | 0.46 | 1.6% | 0.57 | 1.24 ± 0.20 | | 1.14 ± 0.07 | 0.65 | 1.1% | 1.33 | 2.05 ± 0.42 |
|  | d18:0/20:1 | 594.5 | 36.3 |  | N.D. | N/A | N.D. | N.D. | N.D. | N.Q. | N/A | N.Q. | N.Q. | | N.Q. | N.D. | N/A | N.D. | N.D. |
|  | d18:1/22:5 | 612.5 | 34.5 |  | 1.88 | 5.2% | 0.95 | 0.50 ± 0.03 | 0.49 ± 0.01** | 0.33 | 1.1% | 0.32 | 0.97 ± 0.08 | | 0.96 ± 0.05 | 2.14 | 3.5% | 4.45 | 2.08 ± 0.10* |
|  | d18:1/22:3 | 616.5 | 36.3 |  | 1.85 | 5.1% | 0.92 | 0.50 ± 0.02 | 0.52 ± 0.01** | N.Q. | N/A | N.Q. | N.Q. | | N.Q. | N.Q. | N/A | N.Q. | N.Q. |
|  | d18:1/22:1 | 620.5 | 38.6 | **4** | 2.58 | 7.1% | 1.82 | 0.70 ± 0.03 | 0.73 ± 0.01** | 3.40 | 11.9% | 3.31 | 0.97 ± 0.03 | | 0.98 ± 0.04 | 3.66 | 6.0% | 4.20 | 1.15 ± 0.08 |
|  | d18:1/22:0 | 622.5 | 39.6 |  | 0.56 | 1.5% | 0.52 | 0.93 ± 0.04 | 1.00 ± 0.03 | 0.56 | 1.9% | 0.74 | 1.33 ± 0.07 | | 1.33 ± 0.07** | 1.32 | 2.2% | 1.29 | 0.98 ± 0.15 |
|  | d18:1/24:1 | 648.5 | 42.3 | **5** | 11.28 | 31.0% | 5.72 | 0.50 ± 0.04 | 0.48 ± 0.01** | 5.67 | 19.8% | 6.43 | 1.13 ± 0.07 | | 1.17 ± 0.05** | 36.12 | 58.9% | 36.61 | 1.01 ± 0.14 |
|  | d18:1/24:0 | 650.6 | 45.4 |  | 3.24 | 8.9% | 2.03 | 0.62 ± 0.02 | 0.64 ± 0.02** | 1.74 | 6.1% | 1.96 | 1.13 ± 0.14 | | 1.10 ± 0.08** | 3.29 | 5.4% | 2.68 | 0.82 ± 0.07* |
| **MHC** | d18:1/18:0 | 728.5 | 32.5 |  | 2.00 | 1.3% | 1.82 | 0.91 ± 0.06 | 0.91 ± 0.02 | 1.62 | 1.1% | 1.81 | 1.12 ± 0.12 | | 1.38 ± 0.06 | 0.37 | 0.6% | 0.77 | 2.08 ± 0.38* |
|  | d18:1/20:1 | 754.5 | 37.2 |  | 2.20 | 1.4% | 1.92 | 0.87 ± 0.02 | 0.83 ± 0.02** | 2.28 | 1.6% | 4.12 | 1.81 ± 0.29 | | 2.06 ± 0.13** | 0.41 | 0.7% | 0.54 | 1.31 ± 0.14 |
|  | d18:1/20:0 | 756.5 | 38.9 |  | 0.98 | 0.6% | 1.02 | 1.04 ± 0.18 | 1.03 ± 0.07 | 3.25 | 2.2% | 3.71 | 1.14 ± 0.34 | | 1.00 ± 0.10 | 0.32 | 0.5% | 0.51 | 1.61 ± 0.09* |
|  | d18:1/22:2 | 780.5 | 37.8 |  | 7.06 | 4.5% | 6.77 | 0.96 ± 0.03 | 1.00 ± 0.03 | 1.96 | 1.4% | 3.24 | 1.65 ± 0.13 | | 1.61 ± 0.08** | 0.28 | 0.5% | 0.55 | 1.94 ± 0.11** |
|  | d18:1/22:1 | 782.5 | 39.1 | **1** | 10.77 | 6.8% | 9.51 | 0.88 ± 0.02 | 0.91 ± 0.03** | 21.59 | 14.9% | 24.11 | 1.12 ± 0.19 | | 1.18 ± 0.06** | 4.45 | 7.1% | 8.76 | 1.97 ± 0.12** |
|  | d18:1/22:0 | 784.5 | 39.5 |  | 7.56 | 4.8% | 13.41 | 1.77 ± 0.11 | 1.69 ± 0.05** | 5.81 | 4.0% | 12.63 | 2.17 ± 0.35 | | 2.09 ± 0.14** | 3.70 | 5.9% | 7.29 | 1.97 ± 0.16* |
|  | d18:1/24:6 | 800.5 | 33.7 |  | 8.88 | 5.6% | 6.87 | 0.77 ± 0.03 | 0.79 ± 0.02** | 9.05 | 6.2% | 14.37 | 1.59 ± 0.29 | | 1.73 ± 0.10** | 3.37 | 5.4% | 5.09 | 1.51 ± 0.14* |
|  | d18:1/24:2 | 808.5 | 38.7 | **2** | 18.77 | 11.9% | 11.89 | 0.63 ± 0.07 | 0.66 ± 0.02** | 22.89 | 15.8% | 36.66 | 1.60 ± 0.24 | | 1.42 ± 0.09** | 1.96 | 3.2% | 2.61 | 1.33 ± 0.12* |
|  | d18:1/24:1 | 810.5 | 40.4 | **3** | 63.90 | 40.4% | 35.05 | 0.55 ± 0.03 | 0.57 ± 0.02** | 52.80 | 36.4% | 59.74 | 1.13 ± 0.18 | | 1.08 ± 0.08 | 33.92 | 54.5% | 42.18 | 1.24 ± 0.10* |
|  | d18:1/24:0 | 812.5 | 42.3 | **4** | 36.02 | 22.8% | 35.24 | 0.98 ± 0.13 | 1.01 ± 0.03 | 23.90 | 16.5% | 42.50 | 1.78 ± 0.24 | | 1.53 ± 0.10** | 13.48 | 21.6% | 22.75 | 1.69 ± 0.20** |
| **ST** | d18:1/16:0 | 780.5 | 26.1 |  | 0.08 | 4.1% | 0.08 | 1.01 ± 0.03 | 1.05 ± 0.03 | 0.07 | 2.4% | 0.07 | 0.96 ± 0.28 | | 1.01 ± 0.06 | 0.07 | 3.5% | 0.10 | 1.31 ± 0.03** |
|  | d18:1/18:0 | 808.5 | 29.1 | **1** | 0.55 | 27.1% | 0.52 | 0.95 ± 0.07 | 0.98 ± 0.03 | 0.48 | 16.6% | 0.64 | 1.34 ± 0.11 | | 1.27 ± 0.05** | 0.44 | 20.9% | 0.46 | 1.04 ± 0.05 |
|  | d18:1/20:0 | 836.5 | 30.2 |  | 0.05 | 2.4% | 0.04 | 0.91 ± 0.05 | 0.98 ± 0.04 | 0.19 | 6.5% | 0.28 | 1.47 ± 0.16 | | 1.47 ± 0.10** | 0.05 | 2.2% | 0.04 | 0.94 ± 0.10 |
|  | d18:0/22:6 | 854.5 | 26.9 |  | N.D. | N/A | N.D. | N.D. | N.D. | 0.05 | 1.6% | 0.03 | 0.69 ± 0.05 | | 1.11 ± 0.04 | N.Q. | N/A | N.Q. | N.Q. |
|  | d18:1/22:1 | 862.5 | 30.0 |  | 0.20 | 10.1% | 0.20 | 0.96 ± 0.08 | 1.02 ± 0.03 | 0.19 | 6.7% | 0.29 | 1.52 ± 0.05 | | 1.21 ± 0.06 | 0.14 | 6.8% | 0.15 | 1.07 ± 0.07 |
|  | d18:1/22:0 | 864.5 | 30.6 |  | N.Q. | N/A | N.Q. | N.Q. | N.Q. | 0.18 | 6.2% | 0.17 | 0.97 ± 0.14 | | 1.18 ± 0.07 | 0.09 | 4.2% | 0.06 | 0.72 ± 0.06 |
|  | d18:0/22:0 | 866.5 | 31.0 |  | N.Q. | N/A | N.Q. | N.Q. | N.Q. | N.D. | N/A | N.D. | N.D. | | N.D. | N.Q. | N/A | N.Q. | N.Q. |
|  | d18:1/24:1 | 890.5 | 32.6 | **2** | 0.76 | 37.9% | 0.92 | 1.21 ± 0.06 | 1.34 ± 0.04** | 1.25 | 43.5% | 1.47 | 1.18 ± 0.18 | | 1.22 ± 0.07** | 0.90 | 42.7% | 0.64 | 0.71 ± 0.05* |
|  | d18:1/24:0 | 892.5 | 32.9 | **3** | 0.37 | 18.3% | 0.22 | 0.61 ± 0.07 | 0.66 ± 0.03** | 0.36 | 12.6% | 0.30 | 0.83 ± 0.14 | | 0.93 ± 0.09 | 0.33 | 15.4% | 0.36 | 1.11 ± 0.12 |
|  | d18:0/24:0 | 894.5 | 33.0 |  | N.Q. | N/A | N.Q. | N.Q. | N.Q. | 0.11 | 3.9% | 0.10 | 0.87 ± 0.15 | | 0.83 ± 0.07 | 0.09 | 4.2% | 0.07 | 0.78 ± 0.10 |
| **TAG** | 42:0 | 740.7 | 48.0 |  | 0.12 | 1.4% | 0.17 | 1.38 ± 0.28 | 1.18 ± 0.04 | 0.07 | 0.7% | 0.06 | 0.84 ± 0.08 | | 0.84 ± 0.05 | 0.37 | 1.2% | 0.24 | 0.65 ± 0.11 |
|  | 44:1 | 766.7 | 48.4 |  | 0.20 | 2.2% | 0.25 | 1.30 ± 0.18 | 1.50 ± 0.06** | 0.09 | 0.9% | 0.09 | 1.02 ± 0.15 | | 0.81 ± 0.04 | 0.46 | 1.4% | 0.38 | 0.82 ± 0.04* |
|  | 44:0 | 768.7 | 52.9 | **1** | 0.20 | 2.3% | 0.26 | 1.31 ± 0.04 | 1.29 ± 0.04** | 0.11 | 1.0% | 0.16 | 1.51 ± 0.08 | | 1.11 ± 0.10 | 1.46 | 4.6% | 0.67 | 0.46 ± 0.14 |
|  | 46:2 | 792.7 | 51.2 | **2** | 0.31 | 3.6% | 0.36 | 1.15 ± 0.16 | 1.20 ± 0.05** | 0.16 | 1.5% | 0.16 | 0.99 ± 0.29 | | 0.87 ± 0.06 | 1.06 | 3.3% | 0.75 | 0.71 ± 0.07 |
|  | 46:1 | 794.7 | 53.6 | **3** | 0.44 | 5.0% | 0.44 | 1.01 ± 0.06 | 1.09 ± 0.03* | 0.23 | 2.2% | 0.35 | 1.50 ± 0.24 | | 1.14 ± 0.08 | 1.62 | 5.1% | 1.06 | 0.66 ± 0.05** |
|  | 46:0 | 796.7 | 55.7 | **4** | 0.45 | 5.1% | 0.47 | 1.05 ± 0.07 | 1.06 ± 0.02 | 0.32 | 3.0% | 0.39 | 1.21 ± 0.12 | | 1.09 ± 0.07 | 1.45 | 4.6% | 1.22 | 0.84 ± 0.03* |
|  | 48:3 | 818.7 | 58.3 |  | 0.21 | 2.3% | 0.19 | 0.93 ± 0.12 | 1.04 ± 0.05 | 0.09 | 0.8% | 0.10 | 1.12 ± 0.18 | | 0.98 ± 0.08 | 0.78 | 2.5% | 0.34 | 0.43 ± 0.05** |
|  | 48:2 | 820.7 | 61.3 | **5** | 0.52 | 5.9% | 0.50 | 0.96 ± 0.09 | 0.98 ± 0.03 | 0.36 | 3.3% | 0.39 | 1.08 ± 0.35 | | 0.86 ± 0.06 | 1.89 | 5.9% | 1.43 | 0.76 ± 0.06* |
|  | 48:1 | 822.7 | 61.3 | **6** | 0.53 | 5.9% | 0.39 | 0.74 ± 0.04 | 0.70 ± 0.03** | 0.31 | 2.9% | 0.39 | 1.27 ± 0.04 | | 1.09 ± 0.05 | 2.45 | 7.7% | 1.46 | 0.60 ± 0.10 |
|  | 48:0 | 824.7 | 65.3 | **7** | 0.75 | 8.5% | 1.04 | 1.39 ± 0.15 | 1.52 ± 0.06** | 0.65 | 6.1% | 0.82 | 1.27 ± 0.12 | | 1.21 ± 0.03** | 1.73 | 5.5% | 1.61 | 0.93 ± 0.06 |
|  | 50:4 | 844.7 | 55.9 |  | 0.03 | 0.3% | 0.03 | 1.00 ± 0.12 | 1.06 ± 0.10 | N.D. | N/A | N.D. | N.D. | | N.D. | 0.07 | 0.2% | 0.04 | 0.54 ± 0.05** |
|  | 50:3 | 846.7 | 57.6 |  | 0.22 | 2.5% | 0.14 | 0.62 ± 0.07 | 0.63 ± 0.03** | 0.11 | 1.0% | 0.12 | 1.14 ± 0.13 | | 0.90 ± 0.05 | 0.70 | 2.2% | 0.71 | 1.02 ± 0.13 |
|  | 50:2 | 848.7 | 58.7 | **8** | 0.34 | 3.9% | 0.16 | 0.47 ± 0.04 | 0.54 ± 0.02** | 0.16 | 1.5% | 0.23 | 1.42 ± 0.13 | | 1.32 ± 0.04** | 0.72 | 2.3% | 0.70 | 0.97 ± 0.15 |
|  | 50:1 | 850.7 | 60.4 | **9** | 0.68 | 7.7% | 0.62 | 0.91 ± 0.05 | 1.03 ± 0.03 | 0.68 | 6.3% | 0.71 | 1.05 ± 0.06 | | 0.97 ± 0.03 | 2.30 | 7.2% | 1.51 | 0.66 ± 0.06 |
|  | 50:0 | 852.7 | 60.6 | **10** | 0.37 | 4.1% | 0.52 | 1.41 ± 0.05 | 1.50 ± 0.05** | 0.46 | 4.3% | 0.53 | 1.16 ± 0.08 | | 1.22 ± 0.06 | 1.65 | 5.2% | 0.56 | 0.34 ± 0.03* |
|  | 52:4 | 872.7 | 56.1 | **11** | 0.17 | 1.9% | 0.36 | 2.09 ± 0.05 | 1.56 ± 0.13** | 0.87 | 8.1% | 0.40 | 0.46 ± 0.09 | | 0.50 ± 0.03** | 0.90 | 2.8% | 0.52 | 0.58 ± 0.05** |
|  | 52:3 | 874.7 | 57.5 | **12** | 0.28 | 3.2% | 0.22 | 0.77 ± 0.04 | 1.07 ± 0.04 | 0.45 | 4.2% | 0.38 | 0.85 ± 0.31 | | 0.84 ± 0.03** | 0.75 | 2.4% | 0.56 | 0.74 ± 0.07 |
|  | 52:2 | 876.7 | 60.5 | **13** | 0.58 | 6.6% | 0.57 | 0.97 ± 0.06 | 1.02 ± 0.04 | 0.71 | 6.6% | 0.67 | 0.94 ± 0.06 | | 0.96 ± 0.03 | 3.63 | 11.4% | 1.32 | 0.36 ± 0.03* |
|  | 52:1 | 878.7 | 60.6 |  | 0.20 | 2.3% | 0.19 | 0.95 ± 0.06 | 1.02 ± 0.04 | 0.23 | 2.1% | 0.21 | 0.93 ± 0.07 | | 0.96 ± 0.04 | 0.73 | 2.3% | 0.29 | 0.40 ± 0.06** |
|  | 52:0 | 880.7 | 65.9 |  | 0.17 | 1.9% | 0.25 | 1.52 ± 0.07 | 1.51 ± 0.04** | 0.31 | 2.9% | 0.32 | 1.01 ± 0.08 | | 1.08 ± 0.05 | 0.18 | 0.6% | 0.15 | 0.82 ± 0.10 |
|  | 54:6 | 896.7 | 56.4 | **14** | 0.52 | 5.9% | 0.67 | 1.29 ± 0.10 | 1.19 ± 0.06 | 1.44 | 13.5% | 0.46 | 0.32 ± 0.06 | | 0.39 ± 0.03** | 1.17 | 3.7% | 0.95 | 0.81 ± 0.05 |
|  | 54:5 | 898.7 | 56.7 | **15** | 0.36 | 4.0% | 0.40 | 1.13 ± 0.14 | 1.09 ± 0.04** | 0.96 | 9.0% | 0.53 | 0.55 ± 0.08 | | 0.54 ± 0.02** | 1.11 | 3.5% | 0.60 | 0.54 ± 0.06** |
|  | 54:4 | 900.7 | 58.1 | **16** | 0.29 | 3.3% | 0.27 | 0.94 ± 0.19 | 1.03 ± 0.06 | 0.76 | 7.1% | 0.39 | 0.51 ± 0.04 | | 0.70 ± 0.03** | 0.70 | 2.2% | 0.52 | 0.74 ± 0.04* |
|  | 54:3 | 902.7 | 60.7 | **17** | 0.44 | 5.0% | 0.44 | 0.99 ± 0.06 | 1.02 ± 0.03 | 0.76 | 7.1% | 0.52 | 0.68 ± 0.06 | | 0.75 ± 0.03** | 2.62 | 8.3% | 1.24 | 0.47 ± 0.06** |
|  | 54:2 | 904.7 | 63.5 |  | 0.15 | 1.7% | 0.15 | 1.02 ± 0.09 | 0.98 ± 0.04 | 0.16 | 1.5% | 0.15 | 0.94 ± 0.10 | | 0.96 ± 0.05 | 0.41 | 1.3% | 0.20 | 0.48 ± 0.08** |
|  | 54:1 | 906.7 | 65.9 |  | 0.18 | 2.0% | 0.11 | 0.59 ± 0.04 | 0.59 ± 0.02** | 0.14 | 1.3% | 0.17 | 1.16 ± 0.27 | | 0.87 ± 0.04 | 0.35 | 1.1% | 0.32 | 0.89 ± 0.05 |
|  | 54:0 | 908.7 | 67.0 |  | N.D. | N/A | N.D. | N.D. | N.D. | N.Q. | N/A | N.Q. | N.Q. | | N.Q. | 0.07 | 0.2% | 0.05 | 0.75 ± 0.09* |
|  | 56:8 | 920.7 | 54.5 |  | N.D. | N/A | N.D. | N.D. | N.D. | N.D. | N/A | N.D. | N.D. | | N.D. | N.Q. | N/A | N.Q. | N.Q. |
|  | 56:7 | 922.7 | 56.5 |  | 0.10 | 1.2% | 0.07 | 0.63 ± 0.06 | 0.73 ± 0.04** | N.Q. | N/A | N.Q. | N.Q. | | N.Q. | N.Q. | N/A | N.Q. | N.Q. |
|  | 56:5 | 926.7 | 59.4 |  | 0.03 | 0.4% | 0.04 | 1.12 ± 0.08 | 1.13 ± 0.05** | 0.03 | 0.3% | 0.02 | 0.72 ± 0.01 | | 0.61 ± 0.03** | N.D. | N/A | N.D. | N.D. |
|  | 56:2 | 932.7 | 65.5 |  | N.Q. | N/A | N.Q. | N.Q. | N.Q. | 0.01 | 0.1% | 0.02 | 2.12 ± 0.39 | | 1.77 ± 0.12** | 0.03 | 0.1% | 0.02 | 0.74 ± 0.06* |
|  | 56:1 | 934.7 | 66.4 |  | N.Q. | N/A | N.Q. | N.Q. | N.Q. | 0.02 | 0.1% | 0.03 | 2.28 ± 0.45 | | 1.85 ± 0.18** | 0.06 | 0.2% | 0.04 | 0.61 ± 0.07* |
|  | 56:0 | 936.7 | 71.6 |  | N.D. | N/A | N.D. | N.D. | N.D. | N.Q. | N/A | N.Q. | N.Q. | | N.Q. | 0.16 | 0.5% | 0.03 | 0.18 ± 0.03* |
|  | 58:8 | 948.7 | 56.7 |  | N.D. | N/A | N.D. | N.D. | N.D. | N.Q. | N/A | N.Q. | N.Q. | | N.Q. | 0.04 | 0.1% | 0.03 | 0.70 ± 0.08 |
|  | 60:12 | 968.7 | 53.8 |  | N.D. | N/A | N.D. | N.D. | N.D. | 0.05 | 0.5% | 0.02 | 0.36 ± 0.04 | | 0.60 ± 0.07 | 0.12 | 0.4% | 0.10 | 0.80 ± 0.17 |

Table S2. Isomeric structure of a) PC, b) PE, and c) TAG species identified.

a) PC

| **Molecular species** | **Acyl chains** | **m/z** | **Molecular species** | **Acyl chains** | **m/z** | **Molecular species** | **Acyl chains** | **m/z** |
| --- | --- | --- | --- | --- | --- | --- | --- | --- |
| 30:0 | 14:0/16:0 | 706.5 | 38:4 | 18:1/20:3 | 810.5 | 40:5 | 20:1/20:4 | 836.5 |
| 32:2 | 16:1/16:1 | 730.5 |  | 18:0/20:4 | 810.5 |  | 18:0/22:5 | 836.5 |
| 32:1 | 16:0/16:1 | 732.5 |  | 16:0/22:4 | 810.5 |  | 16:0/24:5 | 836.5 |
| 32:0 | 16:0/16:0 | 734.5 |  | 18:1/20:3 | 810.5 |  | 18:1/22:4 | 836.5 |
| 34:2 | 16:0/18:2 | 758.5 | 38:3 | 18:0/20:3 | 812.5 | 40:4 | 18:0/22:4 | 838.5 |
|  | 16:1/18:1 | 758.5 |  | 18:1/20:2 | 812.5 |  | 16:0/24:4 | 838.5 |
| 34:1 | 16:1/18:0 | 760.5 |  | 16:0/22:3 | 812.5 |  | 18:0/22:4 | 838.5 |
|  | 16:0/18:1 | 760.5 |  | 18:2/20:1 | 812.5 | 40:3 | 18:0/22:3 | 840.5 |
| 34:0 | 16:0/18:0 | 762.5 | 38:2 | 18:1/20:1 | 814.5 |  | 20:0/20:3 | 840.5 |
| 36:5 | 16:1/20:4 | 780.5 |  | 16:0/22:2 | 814.5 | 40:2 | 18:1/22:1 | 842.5 |
| 36:4 | 16:0/20:4 | 782.5 |  | 18:2/20:0 | 814.5 |  | 16:1/24:1 | 842.5 |
| 36:3 | 18:1/18:2 | 784.5 |  | 18:0/20:2 | 814.5 |  | 20:1/20:1 | 842.5 |
|  | 16:0/20:3 | 784.5 |  | 16:1/22:1 | 814.5 |  | 18:0/22:2 | 842.5 |
| 36:2 | 16:0/20:2 | 786.5 | 38:1 | 18:0/20:1 | 816.5 | 40:1 | 18:1/22:0 | 844.5 |
|  | 18:1/18:1 | 786.5 |  | 18:1/20:0 | 816.5 |  | 16:0/24:1 | 844.5 |
|  | 18:0/18:2 | 786.5 |  | 16:0/22:1 | 816.5 |  | 20:0/20:1 | 844.5 |
| 36:1 | 18:0/18:1 | 788.5 |  | 16:1/22:0 | 816.5 |  | 18:0/22:1 | 844.5 |
|  | 16:0/20:1 | 788.5 | 38:0 | 16:0/22:0 | 818.5 |  | 16:1/24:0 | 844.5 |
| 36:0 | 16:0/20:0 | 790.5 |  | 18:0/20:0 | 818.5 | 40:0 | 16:0/24:0 | 846.5 |
|  | 18:0/18:0 | 790.5 | 40:8 | 18:2/22:6 | 830.5 |  | 20:0/20:0 | 846.5 |
| 38:7 | 16:1/22:6 | 804.5 |  | 20:4/20:4 | 830.5 | 42:10 | 20:4/22:6 | 854.5 |
| 38:6 | 16:0/22:6 | 806.5 | 40:7 | 18:1/22:6 | 832.5 | 42:8 | 20:4/22:4 | 858.5 |
|  | 18:2/20:4 | 806.5 | 40:6 | 18:0/22:6 | 834.5 |  | 20:2/22:6 | 858.5 |
| 38:5 | 18:1/20:4 | 808.5 |  | 20:2/20:4 | 834.5 | 42:2 | 18:1/24:1 | 870.5 |
|  | 16:0/22:5 | 808.5 |  | 18:2/22:4 | 834.5 | 42:1 | 18:1/24:0 | 872.5 |
|  | 18:2/20:3 | 808.5 |  | 18:1/22:5 | 834.5 |  | 18:0/24:1 | 872.5 |
|  | 16:1/22:4 | 808.5 |  |  |  | 44:12 | 22:6/22:6 | 878.5 |
|  |  |  |  |  |  |  |  |  |

b) PE

| **Molecular species** | **Acyl chains** | **m/z** | **Molecular species** | **Acyl chains** | **m/z** |
| --- | --- | --- | --- | --- | --- |
| 32:1 | 16:1/16:0 | 688.5 | 38:3 | 20:3/18:0 | 768.5 |
| 34:2 | 18:1/16:1 | 714.5 |  | 16:0/22:3 | 768.5 |
| 34:1 | 18:1/16:0 | 716.5 | 38:2 | 18:1/20:1 | 770.5 |
|  | 16:1/18:0 | 716.5 | 38:1 | 18:0/20:1 | 772.5 |
| 34:0 | 16:0/18:0 | 718.5 |  | 18:1/20:0 | 772.5 |
| 36:5 | 20:4/16:1 | 736.5 | 40:7 | 22:6/18:1 | 788.5 |
| 36:4 | 20:4/16:0 | 738.5 | 40:6 | 18:0/22:6 | 790.5 |
| 36:3 | 18:1/18:2 | 740.5 | 40:5 | 22:5/18:0 | 792.5 |
|  | 16:0/20:3 | 740.5 |  | 20:4/20:1 | 792.5 |
| 36:2 | 18:1/18:1 | 742.5 |  | 18:1/22:4 | 792.5 |
| 36:1 | 18:1/18:0 | 744.5 |  | 22:4/18:1 | 792.5 |
|  | 16:0/20:1 | 744.5 | 40:4 | 22:4/18:0 | 794.5 |
| 38:6 | 22:6/16:0 | 762.5 |  | 20:4/20:0 | 794.5 |
|  | 20:4/18:2 | 762.5 | 40:3 | 22:3/18:0 | 796.5 |
| 38:5 | 20:4/18:1 | 764.5 | 42:6 | 18:0/24:6 | 818.5 |
|  | 16:0/22:5 | 764.5 | 42:4 | 24:4/18:0 | 822.5 |
| 38:4 | 20:4/18:0 | 766.5 | 44:10 | 22:6/22:4 | 838.5 |
|  | 16:0/22:4 | 766.5 |  |  |  |
|  | 22:4/16:0 | 766.5 |  |  |  |

c) TAG

| **Molecular species** | **Acyl chains** | **m/z** | **Molecular species** | **Acyl chains** | **m/z** | **Molecular species** | **Acyl chains** | **m/z** |
| --- | --- | --- | --- | --- | --- | --- | --- | --- |
| 42:0 | 14:0/14:0/14:0 | 740.7 | 50:3 | 16:1/16:1/18:1 | 846.7 | 54:3 | 18:1/18:1/18:1 | 902.7 |
| 44:1 | 14:0/14:0/16:1 | 766.7 |  | 14:0/18:1/18:2 |  |  | 18:0/18:1/18:2 |  |
|  | 12:0/16:0/16:1 |  |  | 14:1/18:1/18:1 |  | 54:2 | 18:0/18:1/18:1 | 904.7 |
|  | 14:0/14:1/16:0 |  |  | 16:0/16:1/18:2 |  | 54:1 | 18:0/18:0/18:1 | 906.7 |
| 44:0 | 14:0/14:0/16:0 | 768.7 | 50:2 | 16:0/16:1/18:1 | 848.7 |  | 16:0/18:1/20:0 |  |
|  | 12:0/16:0/16:0 |  |  | 14:0/18:1/18:1 |  |  | 16:0/18:0/20:1 |  |
| 46:2 | 12:0/16:0/18:2 | 792.7 |  | 16:0/16:0/18:2 |  |  | 16:1/18:0/20:0 |  |
|  | 12:0/16:1/18:1 |  | 50:1 | 16:0/16:0/18:1 | 850.7 | 54:0 | 18:0/18:0/18:0 | 908.7 |
|  | 14:0/14:0/18:2 |  |  | 16:0/16:1/18:0 |  |  | 16:0/18:0/20:0 |  |
|  | 14:0/14:1/18:1 |  | 50:0 | 16:0/16:0/18:0 | 852.7 |  | 14:0/18:0/22:0 |  |
|  | 14:0/16:1/16:1 |  | 52:4 | 16:1/18:1/18:2 | 872.7 | 56:8 | 16:1/18:1/22:6 | 920.7 |
|  | 14:1/16:0/16:1 |  |  | 16:0/18:2/18:2 |  | 56:7 | 16:0/18:1/22:6 | 922.7 |
| 46:1 | 14:0/16:0/16:1 | 794.7 |  | 16:0/18:1/18:3 |  | 56:5 | 18:0/18:1/20:4 | 926.7 |
|  | 12:0/16:0/18:1 |  | 52:3 | 18:1/18:1/16:1 | 874.7 | 56:2 | 18:1/18:1/20:0 | 932.7 |
|  | 12:0/16:1/18:0 |  |  | 18:1/18:2/16:0 |  |  | 16:1/20:1/20:0 |  |
|  | 14:0/14:0/18:1 |  |  | 16:0/16:0/20:3 |  |  | 16:1/16:1/24:0 |  |
|  | 14:0/14:1/18:0 |  | 52:2 | 16:0/18:1/18:1 | 876.7 |  | 16:0/20:1/20:1 |  |
|  | 14:1/16:0/16:0 |  |  | 16:0/18:0/18:2 |  | 56:1 | 16:0/16:1/24:0 | 934.7 |
| 46:0 | 14:0/16:0/16:0 | 796.7 |  | 16:1/18:0/18:1 |  |  | 16:0/16:0/24:1 |  |
| 48:3 | 16:1/16:1/16:1 | 818.7 | 52:1 | 16:0/18:0/18:1 | 878.7 |  | 16:0/18:0/22:1 |  |
| 48:2 | 16:0/16:1/16:1 | 820.7 |  | 16:0/16:0/20:1 |  |  | 16:0/18:1/22:0 |  |
|  | 14:0/16:1/18:1 |  |  | 16:1/18:0/18:0 |  |  | 16:1/18:0/22:0 |  |
|  | 12:0/18:1/18:1 |  | 52:0 | 16:0/18:0/18:0 | 880.7 | 56:0 | 16:0/16:0/24:0 | 936.7 |
|  | 14:0/16:0/18:2 |  | 54:6 | 18:2/18:2/18:2 | 896.7 |  | 14:0/18:0/24:0 |  |
|  | 14:1/16:0/18:1 |  |  | 18:1/18:2/18:3 |  |  | 14:0/20:0/22:0 |  |
|  | 16:0/16:1/16:1 |  |  | 16:0/16:0/22:6 |  |  | 16:0/18:0/22:0 |  |
| 48:1 | 16:0/16:0/16:1 | 822.7 | 54:5 | 18:2/18:2/18:1 | 898.7 |  | 16:0/20:0/20:0 |  |
|  | 14:0/16:0/18:1 |  |  | 16:0/18:1/20:4 |  |  | 18:0/18:0/20:0 |  |
|  | 14:0/16:1/18:0 |  | 54:4 | 18:2/18:1/18:1 | 900.7 | 58:8 | 18:1/18:1/22:6 | 948.7 |
| 48:0 | 16:0/16:0/16:0 | 824.7 |  | 18:0/18:2/18:2 |  | 60:12 | 16:0/22:6/22:6 | 968.7 |
| 50:4 | 12:0/18:0/20:4 | 844.7 |  | 16:0/18:0/20:4 |  |  |  |  |
|  | 14:0/16:0/20:4 |  |  |  |  |  |  |  |

Table S3. Type of precursor ion, product ion, and collision energy for each lipid class utilized for targeted quantification with SRM. The number of identified and quantified lipid species with the total amount in each class are listed.

|  |  |  |  | collision | cortex |  |  | hippocampus | |  | hypothalamus | |  |
| --- | --- | --- | --- | --- | --- | --- | --- | --- | --- | --- | --- | --- | --- |
| Class | Precursor ion | Product Ion | | energy (V) | # ID | # Quan. | total level | # ID | # Quan. | total level | # ID | # Quan. | total level |
| LPC | [M+H]^+^ | ^a^[Pcho+H]^+^ | | 25 | 12 | 12 | 2.8 | 12 | 12 | 3.2 | 12 | 12 | 4.5 |
| PC | [M+H]^+^ | ^a^[Pcho+H]^+^ | | 40 | 80 | 35 | 121.3 | 80 | 35 | 84.2 | 80 | 35 | 122.4 |
| LPE | [M+H]^+^ | ^b^[M+H-141]^+^ | | 20 | 12 | 10 | 6.5 | 15 | 13 | 7.2 | 15 | 13 | 6.4 |
| PE | [M+H]^+^ | ^b^[M+H-141]^+^ | | 20 | 36 | 23 | 310.2 | 36 | 23 | 405.3 | 36 | 23 | 387.8 |
| PEp | [M+H]^+^ | ^c^[M+H-RCOOCH_2_CHCH_2_OH]^+^ | | 20 | 35 | 29 | 169.1 | 39 | 39 | 248.8 | 46 | 40 | 203.5 |
| LPG | [M-H]- | [RCOO]^-^ | | 35 | 7 | 6 | 0.1 | 8 | 7 | 0.1 | 9 | 7 | 0.1 |
| PG | [M-H]- | [RCOO]^-^ | | 35 | 19 | 18 | 1.5 | 19 | 19 | 1.6 | 23 | 21 | 1.0 |
| LPI | [M-H]- | [RCOO]^-^ | | 50 | 5 | 5 | 0.1 | 5 | 5 | 0.1 | 7 | 6 | 0.1 |
| PI | [M-H]- | [RCOO]^-^ | | 50 | 19 | 18 | 14.0 | 18 | 18 | 18.1 | 19 | 18 | 17.5 |
| LPS | [M-H]- | [M-H-RCOOH]- | | 35 | 11 | 10 | 0.8 | 11 | 11 | 0.8 | 10 | 10 | 0.6 |
| PS | [M-H]- | [RCOO]^-^ | | 35 | 34 | 25 | 118.6 | 27 | 27 | 79.9 | 32 | 29 | 78.2 |
| LPA | [M-H]- | [M-H-RCOOH]- | | 25 | 10 | 10 | 6.5 | 10 | 10 | 4.2 | 10 | 10 | 4.0 |
| PA | [M-H]- | [RCOO]^-^ | | 25 | 15 | 11 | 0.7 | 9 | 9 | 0.5 | 15 | 13 | 0.6 |
| SM | [M+H]^+^ | ^a^[Pcho+H]^+^ | | 40 | 14 | 14 | 96.6 | 14 | 13 | 37.4 | 14 | 14 | 107.8 |
| Cer | [M+H]^+^ | ^d^[d18:1]^+^ & ^e^[d18:0] | | 30 | 13 | 11 | 36.3 | 13 | 11 | 28.7 | 14 | 11 | 61.3 |
| MHC | [M+H]^+^ | ^d^[d18:1]^+^ | | 40 | 10 | 10 | 158.1 | 10 | 10 | 145.2 | 10 | 10 | 62.3 |
| ST | [M+H]^+^ | ^d^[d18:1]^+^ & ^e^[d18:0] | | 50 | 9 | 6 | 2.0 | 9 | 9 | 2.9 | 10 | 8 | 2.1 |
| TAG | [M+NH4]^+^ | [M+NH_4_-RCOONH_4_]^+^ | | 25 | 81 | 28 | 8.8 | 90 | 29 | 10.7 | 93 | 32 | 31.7 |
| Total |  |  |  |  | 422 | 281 | 1054.0 | 425 | 300 | 1078.9 | 455 | 312 | 1091.9 |

^a^ protonated phosphocholine, ^b^ loss of phosphoethanolamine, ^c^ loss of carboxylate fatty acid in sn-2 position with glycerol backbone, ^d^ sphingosine long chain based fragment, ^e^ sphinganine long chain based fragment

Table S4. List of internal standards and the precursor ion type utilized in targeted quantification.

| Class | Molecular Species | Detected ion | m/z |
| --- | --- | --- | --- |
| LPC | 17:0 | [M+H]^+^ | 510.5 |
| PC | 13:0/13:0 | [M+H]^+^ | 650.5 |
| LPE | 17:1 | [M+H]^+^ | 466.5 |
| PE | 17:0/17:0 | [M+H]^+^ | 720.5 |
| LPG | 17:1 | [M-H]- | 495.5 |
| PG | 15:0/15:0 | [M-H]- | 693.5 |
| PI | 17:0/20:4 | [M-H]- | 871.5 |
| LPS | 17:1 | [M-H]- | 508.5 |
| PS | 17:0/20:4 | [M-H]- | 796.5 |
| LPA | 17:0 | [M-H]- | 421.5 |
| PA | 17:0/17:0 | [M-H]- | 675.5 |
| SM | d18:1/17:0 | [M+H]^+^ | 717.5 |
| Cer | d18:1/17:0 | [M+H]^+^ | 552.5 |
| MHC | d18:1/17:0 | [M+H]^+^ | 714.5 |
| ST | d18:1/17:0 | [M+H]^+^ | 794.5 |
| TAG | 17:0/17:1/17:0 D_5_ | [M+NH_4_]^+^ | 870.7 |
